# Two common, often coexisting grassland plant species differ in their evolutionary potential in response to experimental drought

**DOI:** 10.1002/ece3.10430

**Published:** 2023-08-31

**Authors:** Anna‐Maria Madaj, Walter Durka, Stefan G. Michalski

**Affiliations:** ^1^ Department of Community Ecology Helmholtz‐Centre for Environmental Research – UFZ Halle (Saale) Germany; ^2^ German Centre for Integrative Biodiversity Research (iDiv) Halle‐Jena‐Leipzig Leipzig Germany; ^3^ Institute of Biology Leipzig University Leipzig Germany

**Keywords:** drought, evolutionary potential, genetic variance–covariance matrix, phenotypic response, plants, response to selection

## Abstract

For terrestrial plant communities, the increase in frequency and intensity of drought events is considered as one of the most severe consequences of climate change. While single‐species studies demonstrate that drought can lead to relatively rapid adaptive genetic changes, the evolutionary potential and constraints to selection need to be assessed in comparative approaches to draw more general conclusions. In a greenhouse experiment, we compare the phenotypic response and evolutionary potential of two co‐occurring grassland plant species, *Bromus erectus* and *Trifolium pratense*, in two environments differing in water availability. We quantified variation in functional traits and reproductive fitness in response to drought and compared multivariate genetic variance–covariance matrices and predicted evolutionary responses between species. Species showed different drought adaptation strategies, reflected in both their species‐specific phenotypic plasticity and predicted responses to selection indicating contrasting evolutionary potential under drought. In *T. pratense* we found evidence for stronger genetic constraints under drought compared to more favourable conditions, and for some traits plastic and predicted evolutionary responses to drought had opposing directions, likely limiting the potential for adaptive change. Our study contributes to a more detailed understanding of the evolutionary potential of species with different adaptive strategies in response to climate change and may help to inform future scenarios for semi‐natural grassland ecosystems.

## Graphical abstract

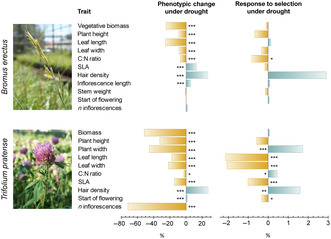

## 1 INTRODUCTION

Climate change is one of the main drivers of biodiversity loss as it leads to rapidly changing environmental conditions on global and local scales (Anderson & Song, 2020; Cahill et al., 2013; IPCC, 2014). For terrestrial ecosystems the increase in the frequency and intensity of drought events is considered to have among the most severe consequences (Knapp et al., 2015; Siepielski et al., 2017). In Europe, semi‐natural grasslands require special attention in biodiversity conservation, being one of the most heterogeneous and species rich terrestrial ecosystems (Isselstein et al., 2005). Extensively managed grasslands are already threatened by land‐use intensification since the beginning of the 20th century (Hejcman et al., 2013) and climatic extremes such as drought may add to and worsen this situation since for many grassland species reduced precipitation is already present all over their natural range (Wellstein et al., 2017). Hence, studies investigating the ability of grassland species to cope with predicted precipitation deficits are urgently needed to protect the biodiversity and associated ecosystem functions of European grasslands (Cahill et al., 2013; Visser, 2008).

The potential for evolutionary adaptation of local populations in response to environmental changes is captured by the additive genetic variance in fitness (Etterson, 2004; Fisher, 1930). This, in turn, is affected by three interacting factors (Fordyce, 2006): The degree of intraspecific genetic variation providing the fundamental source for evolutionary change, the ability of this genetic variation to express different phenotypes (Ghalambor et al., 2007) and lastly, the strength and direction of selection which promotes evolutionary changes. Environmental conditions such as drought are very likely to affect the potential for evolutionary responses as it may influence both the expression of genetic variance in quantitative traits (cf. Stojanova et al., 2019) as well as the strength and direction of selection (Chevin et al., 2010). Additionally, phenotypic plasticity as an important short‐term response mechanism to environmental changes may interact with the evolutionary potential in either direction, promoting or hindering adaptive responses via, for example co‐ and counter‐gradient plasticity or maladaptation (Ghalambor et al., 2007; Merilä & Hendry, 2014).

While there is abundant evidence from single‐species studies that climate change can result in relatively rapid adaptive genetic changes (e.g. Franks et al., 2007; Kovach et al., 2012; Ravenscroft et al., 2015; Warwell & Shaw, 2019), understanding the evolutionary potential and selection constraints to drought for additional, including co‐occurring species, and thus ultimately on community level, remains a major challenge (Chevin et al., 2013; Franks et al., 2014) because of the huge experimental effort.

Phenotypic plasticity and evolutionary responses to drought are likely to affect a species' functional traits, which in turn, for example in grasslands, may impact on community functions such as quality and quantity of biomass production. Three classical adaptive mechanisms exhibited by plants to cope with drought have been defined: (i) tolerance, (ii) avoidance, and (iii) escape (Ludlow, 1989). Each is characterised by a particular combination of traits that lead to a specific phenotype during drought (Kimball et al., 2017; Volaire, 2018). However, plants have a wide spectrum of drought responses and not all species fit perfectly in a particular, or only one, of these drought response categories (Chapman & Auge, 1994; Jones, 1992; Kimball et al., 2017).

Here, following a comparative approach, we quantify genetic covariances between traits and fitness examining the evolutionary potential in response to drought for *Bromus erectus* Huds. (Poaceae) and *Trifolium pratense* L. (Fabaceae). Both species are native perennial, often co‐occurring and common herbs in central European grasslands. Whereas *B*. *erectus* has been described as a drought‐tolerant species which can cope with high rates of dehydration and shows a high survival after severe drought events (Pérez‐Ramos et al., 2013), the important forage crop *T*. *pratense* is known to be sensitive to soil water deficits and susceptible to severe drought events (Hofer et al., 2016; Peterson et al., 1992). However, apart from this phenotypic plasticity, the impact of drought on genetic trait variance, selection constraints and thus, the evolutionary potential in response to drought is still unknown for both species. Assuming drought to be a stronger environmental stressor for *T*. *pratense* than for *B*. *erectus*, we hypothesise also stronger responses to selection for the former (Hoffmann & Hercus, 2000).

Using a greenhouse common garden experiment, we specifically ask how (1) observed phenotype, (2) the expression of multivariate genetic trait variance and selection constraints, and (3) predicted evolutionary responses differ between experimental conditions (control and drought) and species. Answering these questions will gain a more detailed understanding of the evolutionary potential of species with different adaptive mechanisms in response to climate change eventually help to inform future scenarios for semi‐natural grassland ecosystems (cf. Hetzer et al., 2021).

## 2 MATERIALS AND METHODS

### 2.1 Study site and species

We established a greenhouse common garden experiment in the experimental field station Bad Lauchstädt near Halle (Saale), Saxony‐Anhalt, Germany (51.392°N, 11.876°E, 116 m a.s.l.). Here, we examined *B. erectus* Huds. (Poaceae) and *T. pratense* L. (Fabaceae). Both species are perennial outcrossers, frequently coexisting in the field (e.g. on calcareous grasslands; Bonanomi et al., 2012; Thürig et al., 2003) and part of commercially available regional seed mixtures (Rieger‐Hofmann GmbH, Saaten Zeller GmbH & Co. KG).

In seed selection we follow the context of ecological restoration and, in particular, the ‘Regional Admixture Provenancing’ approach, in which seeds from multiple local source populations are used for restoration in order to preserve local adaptation as well as to achieve high levels of intraspecific variation as fundamental source for evolutionary changes (Bucharova et al., 2018). Hence, seed material for each species was sourced from multiple natural populations, that is five for *B. erectus* and four for *T. pratense* originating from an environmentally homogeneous, regional area in Central Germany with maximum distance between source populations of 127 and 119 km for *B. erectus* and *T. pratense*, respectively (Table S1). Seeds were provided through a company specialised in production of regional seeds (Saale Saaten), which holds permissions for seed collections.

In order to minimise the bias of maternal carryover effects on total phenotypic variation (Roach & Wulff, 1987), we first grew an F0‐generation for *B. erectus* (2016) and *T. pratense* (2018) as described in Madaj et al. (2020). F1‐seed families, that is seeds from an open‐pollinated maternal individual, were collected for *B. erectus* in the second growing season (2017, because not all plants flowered in the first growing season) and for *T. pratense* in the first growing season (2018). As both species are obligatory outcrossing and self‐incompatible, we assume seeds mostly to be maternal half siblings and hence variance components explained by seed family to represent mainly the evolutionary relevant additive genetic variance (Falconer & Mackay, 1996). Seeds were stored in a dry place at room temperature (20–25°C).

### 2.2 Experimental design

The experiment was established using seeds from 103 to 104 half‐sib seed families, for *B. erectus* and *T. pratense*, respectively. In February (2018 – *B. erectus*; 2019 – *T. pratense*) ten individuals per seed family were germinated and pricked as described in Madaj et al. (2020). Afterwards, 1030 individuals for *B. erectus* and 1040 individuals for *T. pratense* were potted individually into three‐litre plastic pots, containing about 1.5 kg of a peat free soil–sand substrate (3:1). Plants were watered equally on demand for 8 weeks. In April, pots were placed in a greenhouse in Bad Lauchstädt with ambient temperature and light conditions. For each species, individuals were arranged in five blocks with two individuals per seed family in each block but otherwise random location within blocks. Consistent with expected regional future climatic conditions predicting stronger drought events in summer (Schädler et al., 2019), the experimental treatment started immediately with the finalisation of the establishment phase 10 weeks after germination. Here, one individual per seed family and block received the ‘control’ treatment by keeping soil moisture of the pot at 60% of total soil water capacity (Majer, 2008). The other individual was kept under severe drought with no more than 30% of the soil water capacity (Hoffmann, 2010). Soil moisture was checked by weighing pots every 2 days. The treatment was maintained until flowering and subsequent functional trait measurements were completed for both species in August 2019. During the experiment one and two individuals died in *B. erectus* and *T. pratense*, respectively.

### 2.3 Functional traits

For all individuals (total *n* = 2067), we quantified a set of functional traits known to respond to drought. First, we assessed growth related traits as they are known to be linked to competitive ability and tolerance to, or avoidance capacity of, environmental stress (Cornelissen et al., 2003; Gaudet & Keddy, 1988; Moles et al., 2009). In particular, for both species we assessed plant height (cm) as distance between ground level and top end of the tallest inflorescence, and above ground biomass (g), which was directly cut above ground level, oven dried for 48 h at 70°C and weighted subsequently. In our experiment, biomass is directly reflecting water use efficiency (Briggs & Shantz, 1913), as all individuals received the same amount of water per treatment.

Additionally, we quantified the weight of the tallest stem (g) and its inflorescence length (cm) for *B. erectus*. The stem was cut above ground level, oven dried and weighted similarly to above ground biomass. Finally, we quantified the total plant width for *T. pratense* (cm) as distance between stem tips of the longest branches.

Second, leaf related characteristics were quantified as they are considered to be direct indicators for drought stress, due to the fact that they are linked to water availability. For both *B. erectus* and *T. pratense*, we collected three mature, vegetative leaves from each individual, measured leaf dimensions for each leaf and calculated mean values for leaf width (mm) and leaf length (cm). The species are expected to show smaller leaf dimensions with decreasing water availability. Subsequently, we conducted scans of all leaves and calculated total leaf area using the computer image analysis system WinFOLIA (Version: 2016b Pro; Regent Instruments Canada Inc.). Leaves were oven dried for 48 h at 70°C, weighted and SLA was calculated as the ratio of leaf area (mm^2^) to dry mass (mg) (Knevel et al., 2005). SLA is presumed to be positively related with potential relative growth rates and shows a negative correlation with drought survival (Bongers et al., 2017). Additionally, we assessed C:N ratios of the leaves for both species by analysing the oven dried leaf material from half of the individuals using an automatic elemental analyser (Vario EL III, Elementar Analysensysteme). Drought can shift plant internal carbon (C) and nitrogen (N) stoichiometry towards both increased and decreased C:N ratios (e.g. Lu et al., 2009; Luo et al., 2017; Sardans et al., 2008; Sun et al., 2020), which may affect, for example the forage quality of extensively managed grasslands (Mattson, 1980). Completing leaf trait analyses, leaf hairiness was assessed as it is known to play an important role in plant protection in response to biotic and abiotic stresses, like drought, radiation, herbivores or pathogens (Roy et al., 1999). For *B. erectus*, we counted hairs along the leaf margin. For *T. pratense*, we estimated the number of abaxial hairs on the leaflets on an ordinal, but approximately linear scale with 10 levels of hairiness (1 low to 10 high).

Third, flowering phenology is known to respond to drought with, for example either earlier flowering (Franks et al., 2007; Kooyers, 2015; Shavrukov et al., 2017) or delayed flowering (Fox, 1990; Pantuwan et al., 2002) as a drought escape mechanism. Hence, we quantified the start of flowering (*d*).

Lastly, we assessed the number of inflorescences for both species as a measure of individual fitness.

For *B. erectus*, vegetative biomass and all leaf related traits were assessed in the first growing season on non‐reproductive individuals (August 2018). Because only a few individuals of *B. erectus* came into flower in 2018, all remaining traits were assessed on reproductive individuals in the second year (May–August 2019). For *T. pratense*, we investigated the entire set of functional traits in the first growing season (June–August 2019).

### 2.4 Data analyses

#### 2.4.1 Plastic response

Analysis of the response of phenotypic traits was conducted using linear and generalised linear mixed effect models implemented in the *lme4* package (Bates et al., 2015). For continuous traits with normally distributed residuals, we implemented ‘linear’ mixed effect models, whereas for count data (i.e. hairs and number of inflorescences) we fitted ‘generalised linear’ mixed effect models with a Poisson distributed error structure. Models were run separately for each trait explaining observed phenotypic variation by ‘treatment’ as fixed and ‘seed family’ and ‘block’ as random effects. Significance of the treatment effect was assessed based on 95% credible intervals for model estimates obtained by 10,000 simulations for each model using the *sim* function in the *arm* package (for more details see: Bucharova et al., 2017; Korner‐Nievergelt et al., 2015). Comparing 95% credible intervals is more reliable for testing significance of fixed factors in ‘generalised linear’ mixed effect models than *p*‐values from classical ANOVA (Bolker et al., 2009). Treatment effects were considered to be significant if credible intervals did not overlap with the other treatment mean. Log‐scaled model outputs from generalised linear mixed effect models were back‐transformed to the observed scales by exponentiation of estimates and quantiles. The overall treatment effect was compared between species by calculating the absolute mean difference standardised by the pooled standard deviation (Cohen's *d*; Cohen, 2013) averaged across traits with confidence intervals obtained by bootstrapping across traits 100 times.

#### 2.4.2 Genetic variance and covariance

We estimated the additive genetic variance–covariance matrix (G) for each treatment and species by first standardising all phenotypic traits by their means following Hansen and Houle (2008). Subsequently, we implemented a multivariate, linear mixed effect model in a Bayesian‐MCMC framework, where individual phenotypic trait combinations were explained by ‘seed family’ as random and ‘block’ as fixed effects (MCMCglmm; Hadfield, 2010). Additive genetic variances (*V*_A_) and covariances within and among traits were then extracted from the model as effects explained by seed family.

For the comparison of G‐matrices, a vast number of methods are available (e.g. Cheverud & Marroig, 2007; Robinson & Beckerman, 2013). Here we use eigenvalues and eigenvectors of G‐matrices to calculate the ‘effective number of dimensions’ (*n*_D_), ‘maximum evolvability’ (*e*_max_) and the total genetic variance (*tgv*) as defined in Kirkpatrick (2009). These parameters sufficiently summarise size, shape and structure of the matrix, are interpretable in the context of selective responses and allow comparisons across traits and species (cf. Pitchers et al., 2014). Second, to compare the genetic architecture across treatments on individual trait level, we converted the genetic covariance matrices into genetic correlation matrices and visualised them with the package *qgraph* (Epskamp et al., 2012).

#### 2.4.3 Evolutionary potential

For each treatment, we first calculated relative individual fitness as the total number of inflorescences divided by the experimental population mean. All other phenotypic traits were centred at zero, that is trait means were set to zero. We implemented multivariate linear mixed effect models as described above and extracted G‐matrices for each treatment. In contrast to G‐matrices above, we here followed the approach of Stinchcombe et al. (2013) by not applying any standardisations of the phenotypic traits which allows us to estimate the response to selection in original units (e.g. biomass in grams, start of flowering in Julian days, etc.).

Predictors of the evolutionary potential on trait level were then calculated from the treatment‐specific G‐matrix (Lande, 1979; Lynch & Walsh, 1998; Stinchcombe et al., 2013) as follows. Heritability was defined as *H* ^2^ = *V*_A_/*V*_P_, with *V*_A_ and *V*_P_ representing additive genetic and total phenotypic trait variation, respectively. *V*_P_, in turn, is given by *V*_P_ = *V*_A_ + *V*_R_, where *V*_R_ equals the residual variance. Genetic covariance between trait and fitness (*s*_g_) and selection gradient *β*, given by *β* = G^−1^*s_g_ (Lande & Arnold, 1983; Rausher, 1992) were calculated separately allowing to distinguish between direct and indirect selection effects. For example, a significant genetic covariance between trait and fitness but a selection gradient not significantly different from zero may indicate indirect selection on the respective trait via genetically correlated traits, rather than direct selection (Stinchcombe et al., 2013). The response to selection, indicating the predicted change in the mean of the phenotypic trait after one generation, was defined as Δ*z* = G*β = *s*_g_ (Lande, 1979). Estimates of *β* and Δ*z* were considered significant when the 95% credible interval of the posterior distribution did not overlap zero.

To best fit the multivariate models, we explored a variety of priors for the residual and random effects to ensure the insensitivity of our results to prior specifications (Table S4). For more details on prior choice, model specifications, and MCMC diagnostics refer to Appendix S1 (incl. text paragraph; Deviance Information Criteria in Table S5; all model fits in Tables S6–S13).

All statistical analyses were performed with R Version 4.0.3 (R Core Team, 2018).

## 3 RESULTS

### 3.1 Plastic response

**FIGURE 1:**
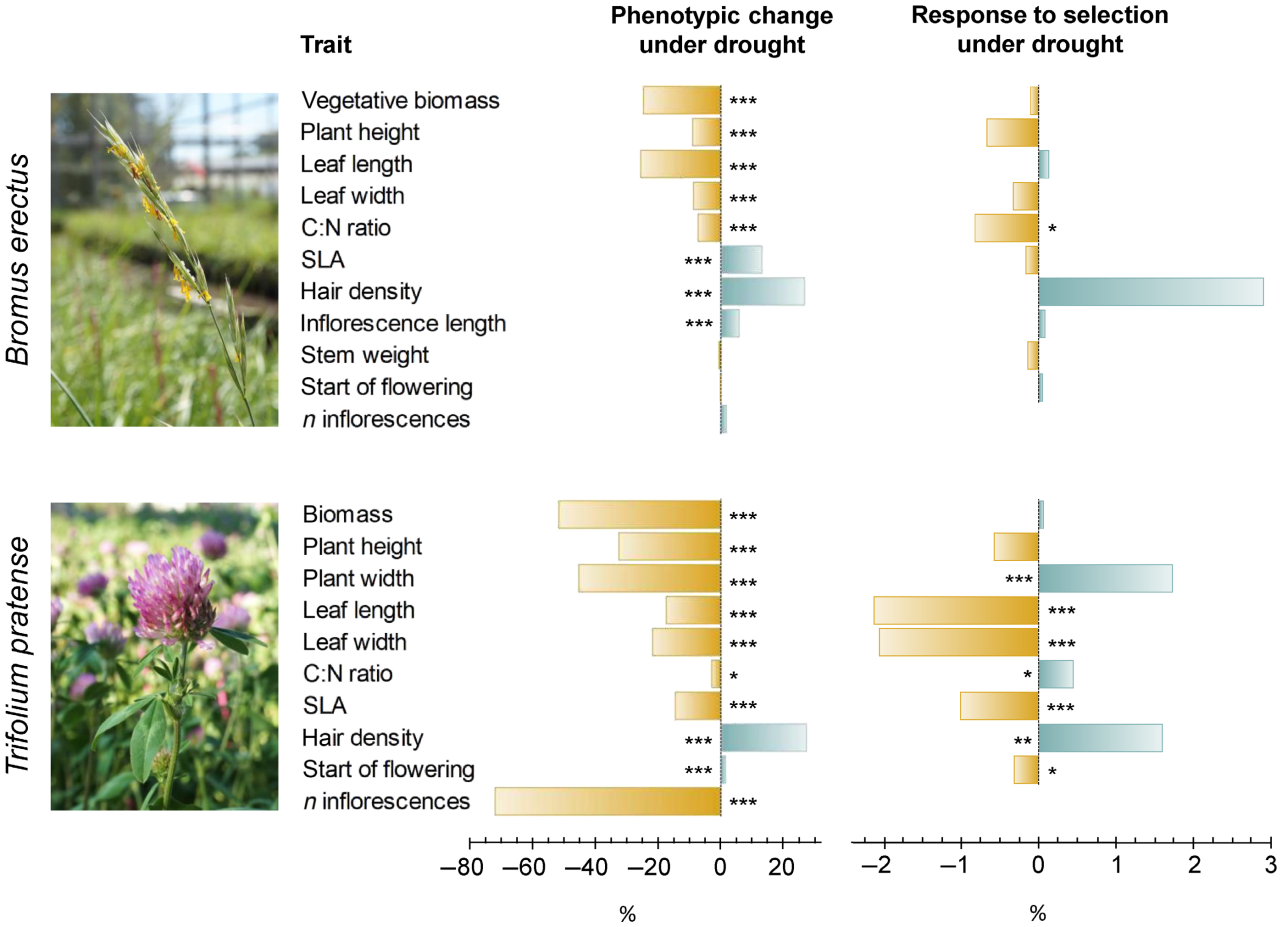
Summary plot. Trait specific phenotypic change under drought compared to control and response to selection under drought, represented as % change compared to mean trait expressions under drought, for *Bromus erectus* and *Trifolium pratense*. Significant treatment effects are marked with asterisks (***p ≤ .001; **p ≤ .01; *p ≤ .05).

Both species revealed drought‐induced phenotypic plasticity (Figure 1, Tables S2 and S3). In general, growth related traits including biomass, plant height and width significantly decreased under drought. Likewise, most leaf traits revealed similar patterns between species. Leaves were significantly smaller, and had lower C:N ratios but were hairier under dry conditions. Interestingly, drought significantly increased SLA in *B. erectus*, but decreased it in *T. pratense*. Flowering start and number of inflorescences revealed no significant treatment effects in *B. erectus* but showed significantly later start of flowering and reduced flower production under drought in *T. pratense*. Across all traits, we found a stronger plastic response to drought in *T. pratense* compared to *B. erectus* (*T. pratense d* = 0.75, CI 0.39–1.21, *B. erectus d* = 0.21, CI 0.10–0.40).

### 3.2 Genetic variance and covariance

**FIGURE 2:**
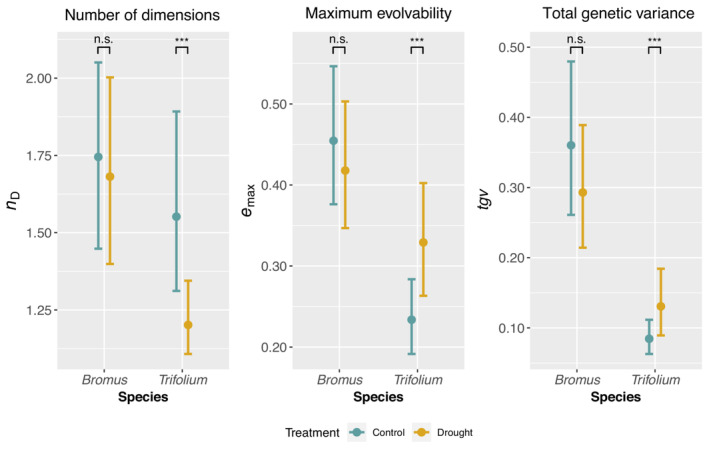
G‐matrix comparison statistics for *Bromus erectus* and *Trifolium pratense*. Patterns of multivariate genetic variance and co‐variance are summarised by the ‘effective number of dimensions’ (*n*_D_), the ‘maximum evolvability’ (*e*_max_) and the ‘total genetic variance’ (*tgv*). Effect sizes for each treatment, that is ‘Control’ and ‘Drought’, are coloured in blue and yellow, respectively. 95% Bayesian credible intervals (CI) are shown as measure of uncertainty. ***Significant treatment effect (CI does not overlap with other treatment mean); n.s., no significant treatment effect.

In *B. erectus*, G‐matrix summary statistics, that is the effective number of dimensions (*n*_D_), the maximum evolvability (*e*_max_) and the total genetic variance (*tgv*), revealed no significant treatment effects (Figure 2, Table S14), indicating that the genetic basis of the multivariate phenotype was not affected by drought. In contrast, drought significantly decreased the effective number of dimensions along with increases in both maximum evolvability and total genetic variance in *T. pratense* (Figure 2, Table S14), suggesting that the genetic basis of the multivariate phenotype is more constrained under drought.

**FIGURE 3:**
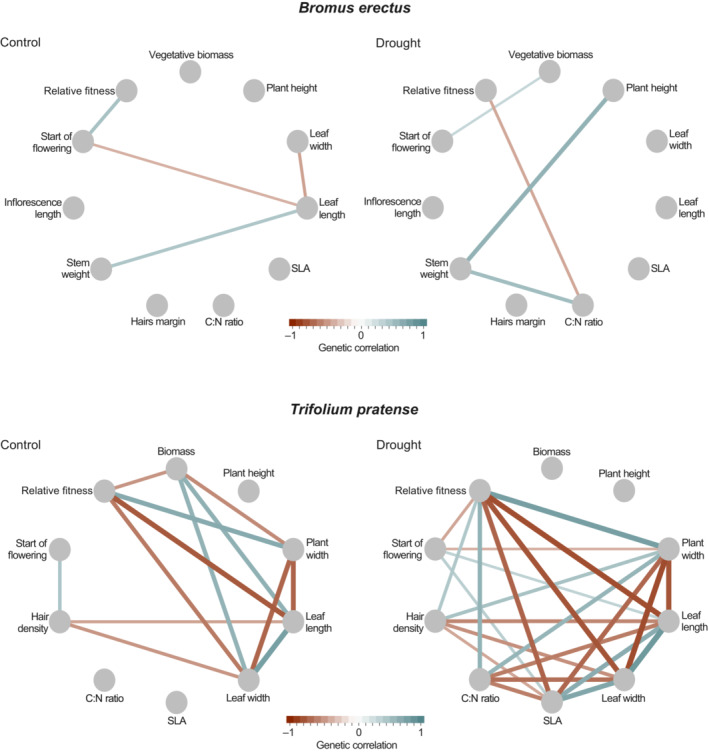
Genetic trait architecture assessed as genetic correlations between quantitative traits for species and treatments. Lines coloured in blue and red indicate significantly positive and negative genetic correlations, respectively. The thickness of the lines represents the strength of correlation.

In *B. erectus*, for both treatments, genetic correlations among traits were significant for only 4 out of 55 possible trait combinations (Figure 3), which does not differ from random expectations (exact binomial test, *p* = .36), yet trait correlations differed across treatments. In *T. pratense*, we found a substantial increase from 13 to 24 significant genetic correlations within the 45 possible trait combinations in response to drought (exact binomial test, *p* < .001, Figure 3).

### 3.3 Predicted evolutionary response

Both species exhibited a similar range of estimated trait heritability regardless of treatment conditions (*H* ^2^ = 0.02–0.34 in *B. erectus*; *H* ^2^ = 0.01–0.39 in *T. pratense*; see Tables S15 and S16). However, treatment specific differences were found for vegetative biomass, leaf width and SLA and for plant width and start of flowering for *B. erectus* and *T. pratense*, respectively.

The response to selection Δ*z* in *B. erectus* predicted evolutionary homeostasis in both treatments for all traits except for two (Figure S1, Table S17). The start of flowering was predicted to shift significantly towards later flowering under control conditions, whereas the mean C:N ratio was predicted to decrease under drought. In contrast, *T. pratense* revealed significant responses to selection in most traits (Figure S1, Table S18). First, a selection towards wider plants with smaller leaf area, that is reduced leaf length and width, was predicted independent of treatment. Second, under control conditions, *T. pratense* revealed a significant response to selection towards reduced biomass. Finally, selection favouring plants to flower earlier, with more hairy leaves and reduced SLA was predicted under drought.

In *B. erectus*, we found neither significant selection gradients nor differences across treatments among all tested functional traits (Table S19). For *T. pratense*, we found ‘leaf length’ to have a significant selection gradient towards producing shorter leaves under control conditions (Table S20).

## 4 DISCUSSION

### 4.1 Plastic response

Overall, *B. erectus* and *T. pratense* revealed different drought adaptation strategies, reflected in both their species‐specific phenotypic plastic and predicted evolutionary responses, indicating contrasting evolutionary potential under drought.

Not unexpected, both species experienced drought‐induced reductions in biomass, plant height (*B. erectus*), plant width (*T. pratense*) and leaf dimensions. All of these traits are known to be closely linked to water availability and water use efficiency, with drought leading to lower growth rates and smaller leaf dimensions (DeWoody et al., 2015; Westoby & Wright, 2006).

Increased leaf hair density and a decreased C:N ratio under drought for both species can be interpreted as passive and/or active consequences of water shortage. If the same number of trichomes per leaf is expressed under drought compared to control conditions, drought‐induced smaller leaves are passively more hairy. This, in turn, may help to reduce water loss from transpiration through an increase of the leaf‐air boundary layer resistance (Galdon‐Armero et al., 2018; Guerfel et al., 2009). However, trichome development may also be actively modulated, for example via differential gene expression (e.g. Ning et al., 2016; Wang et al., 2021).

Likewise, the C:N ratio can be passively reduced under drought, for example via higher N concentrations in leaves as a result of a reduction in biomass, plant and leaf dimensions (Hoang et al., 2019), or actively, by the allocation of C and N into belowground and aboveground biomass, respectively (Weih et al., 2011). At least for *T. pratense*, it has been shown that drought can change gene expression dramatically accompanied by an active increase of several key metabolites in leaves (Yates et al., 2014), probably shifting C:N as a result.

Contrasting patterns between the species were revealed by the plastic response of SLA and reproductive traits. Whereas drought decreased SLA and led to a more belated but strongly reduced reproductive output in *T. pratense*, it increased SLA in *B. erectus* and did not significantly affect reproduction. Specific leaf area is considered to be a direct indicator of drought stress, where the adaptive trade‐offs describe a positive correlation with potential growth rates and a negative correlation with drought survival (Biere, 1996; Scheepens et al., 2010). Hence, our results demonstrate that *T. pratense* follows a drought avoidance strategy by avoiding leaf dehydration, growing slower and reproducing later, all mechanisms known to enhance drought survival (Albert et al., 2010; Jung et al., 2014; Wellstein et al., 2017). In contrast, *B. erectus* increased SLA in response to drought, which is indicative of two other, not necessarily mutually exclusive drought adaptation strategies (Kimball et al., 2017). On the one hand, the increased SLA could point towards drought escape, in which perennial plants cope with soil water limitation by early leaf senescence to become dormant during drought (Blumenthal et al., 2020; Huang, 2008). On the other hand, higher SLA is associated with dehydration tolerance, in which leaf senescence results in the maintenance of turgor stability and growth allocation by increasing root foraging in deeper soil layers. This is in line with Pérez‐Ramos et al. (2013) demonstrating that out of four investigated grassland species, *B. erectus* had highest rates of drought survival and exhibited the deepest root system with highest root elongation rates to tap deep soil water.

### 4.2 Evolutionary potential

#### 4.2.1 Genetic variances and covariances

Comparing the G‐matrices for both species with a meta‐analysis on genetic multivariate trait architecture across plants and animals (Pitchers et al., 2014) showed that all summary parameters (*n*_D_, *e*_max,_ *tgv*) were within the reported 95% credible interval. Specifically, the effective number of dimensions was very similar to the average obtained for many species, and maximum evolvability and total genetic variance were at the lower end of the distribution. Drought did not affect the genetic architecture of the multivariate phenotype in *B. erectus* but shifted it significantly in *T. pratense*, as indicated by changes in the G‐matrix. The effective number of dimensions decreased, indicating a loss of ‘genetic degrees of freedom’ (Kirkpatrick & Lofsvold, 1992; Schluter, 1996), which is also reflected in an increased number of significant among‐trait genetic correlations compared to control conditions. In contrast, total genetic variance and maximum evolvability increased under drought, which could largely be attributed to an increase in the variance of fitness under drought in *T. pratense*. A similar response has been found by Torres‐Martínez et al. (2019), where environmental stress increased the additive genetic variance in fitness for the herb *Lasthenia fremontii*, with the highest effect sizes under dry conditions.

#### 4.2.2 Predicted evolutionary response

Additional to the drought‐induced changes in the G‐matrix, *T. pratense* exhibited more significant trait‐specific predicted evolutionary responses than *B. erectus*. Thus, we will focus on *T. pratense* first. Except for one trait (leaf length in *T. pratense* under control conditions), all significant genetic covariances between trait and fitness could not be attributed directly to the traits analysed as indicated by the non‐significant selection gradients but are probably indirectly driven by genetic covariance with other measured or unmeasured traits (Stinchcombe et al., 2013). For *T. pratense*, the direction of predicted trait‐specific selective responses under drought mirrored the plastic response for SLA, leaf dimensions and hairiness. It could be argued that in these cases, plasticity facilitates adaptive trait changes because it maintains fitness under stressful conditions. In contrast, for plant width, leaf C:N and start of flowering the predicted selective responses to drought had the opposite direction compared to the observed plastic response (Figure 1). Here, the physiological mechanisms underlying the plastic response may, on the one hand, come at the cost of reproductive performance, which in turn may counteract adaptive trait changes, or, on the other hand, facilitate rapid evolution by increasing the strength of selection (Ghalambor et al., 2007; Gibert et al., 2019). Hence, the observed plastic response of *T. pratense* to drought could be non‐ or even mal‐adaptive. This pattern has been described as counter gradient selection, indicating that our prolonged, experimental drought falls outside the environmental conditions to which the regional population of *T. pratense* has adapted to in the past (Gibert et al., 2019). Lastly, one trait, biomass, showed a response to selection only in the control treatment in *T. pratense*. Here, rather counterintuitive and contrasting to the plastic response, selection favours reduced biomass. One reasonable explanation may be nutrient limitation due to the fact that plants were not fertilised additionally while the experiment was running. Hence, the fast‐growing plants under favourable control conditions were most likely limited in nutrients in contrast to plants with reduced growth under water restriction (Fisher et al., 2012).

*Bromus erectus* is known to be well adapted to drought (Grime et al., 2014), which is corroborated by our results. The species remained close to its ecological optimum during drought, with only small changes in fitness‐relevant traits and the overall genetic variance compared to control conditions. Liancourt et al. (2005) demonstrated that out of three investigated co‐occurring grassland species, *B. erectus* was least affected by drought, but most impacted by interspecific competition. Although competition is important in all environments, *B. erectus* may have a significant advantage in the face of climate change. The species is already dominant in conditions of drought and disturbance (Corcket et al., 2003). Predicted increases in the frequency of drought events may rapidly change grassland conditions in a way favourable for the persistence and expansion of *B. erectus* but not for competitors more sensitive to drought (Bradley et al., 2016). Our predictions corroborate demographic analyses of *B. erectus* populations established from the very same seed sources as our experiment, showing significantly positive population growth rates under both ambient as well as future climatic conditions (Lemmer et al., 2021) in grassland plots of the Global Change Experimental Facility GCEF (Schädler et al., 2019).

Understanding the impact of drought events on *T. pratense* is important in particular for agriculture (e.g. Dougherty, 1972; Peterson et al., 1992) where it is in use for crop rotation systems, intercropping or as livestock forage in meadows and pastures. Our findings together with previous results, demonstrating strong genotypic effects in response to drought (Loucks et al., 2018; Yates et al., 2014), are underlining the potential of *T. pratense* to cope with drought on both short‐term and more long‐term temporal scales via plastic and adaptive responses, respectively. However, drought is shifting the species away from its ecological optimum, forcing a change in the genetic basis of the multivariate phenotype and eventually limiting the evolutionary response for some traits.

In controlled environments, like our experiment, observed trait genetic variances and predicted evolutionary responses are likely overestimated relative to natural conditions, where biotic interactions may constrain both and where also selection varies in space and time and across life‐history episodes (Geber & Griffen, 2003). For example, in addition to the expected reduction in the amount of precipitation, future climate scenarios predict increased variability of precipitation events, which may have various results for plant fitness (March‐Salas et al., 2019).

To test for the predictive power of experimental approaches, the evolutionary potential and selective responses need to be assessed in real‐world situations where evolutionary adaptations are actually taking place, that is in the natural habitat (Kruuk et al., 2014).

## AUTHOR CONTRIBUTIONS

**Anna‐Maria Madaj:** Conceptualization (equal); data curation (lead); formal analysis (equal); funding acquisition (equal); investigation (lead); methodology (equal); project administration (equal); resources (equal); software (equal); validation (equal); visualization (lead); writing – original draft (lead); writing – review and editing (equal). **Walter Durka:** Conceptualization (equal); data curation (supporting); formal analysis (supporting); funding acquisition (equal); investigation (supporting); methodology (equal); project administration (equal); resources (equal); software (supporting); supervision (supporting); validation (supporting); visualization (supporting); writing – original draft (supporting); writing – review and editing (equal). **Stefan G. Michalski:** Conceptualization (equal); data curation (supporting); formal analysis (equal); funding acquisition (equal); investigation (supporting); methodology (equal); project administration (equal); resources (equal); software (equal); supervision (lead); validation (equal); visualization (supporting); writing – original draft (supporting); writing – review and editing (equal).

## CONFLICT OF INTEREST STATEMENT

The authors declare no conflict of interest.

## Supporting information


Appendix S1.

## Data Availability

Raw data that support the findings of this study are openly available in *DRYAD* at https://doi.org/10.5061/dryad.cc2fqz6c4. Additional texts, tables and figures are available as Supporting Information.
