# Supplementary material for: Two common, often coexisting grassland plant species differ in their evolutionary potential in response to experimental drought
Source: Ecol Evol. 2023 Aug 31;13(9):e10430. doi: 10.1002/ece3.10430 (PMC10469005; doi:10.1002/ece3.10430)
Supplement: Supplementary file 1 — Appendix S1. [file ECE3-13-e10430-s001.pdf]

SUPPORTING INFORMATION

Two common, often coexisting grassland plant species differ in their evolutionary potential in response to experimental drought

Anna-Maria Madaj, Walter Durka & Stefan G. Michalski

Prior choice, model specifications and MCMC diagnostics

Literature shows manifold possibilities for prior specification (see Bolker et al. 2009, Stinchcombe et al. 2013). We investigated priors where the list of the diagonal elements (V) for the residual and random effects was 0.5 × the phenotypic trait variance (V_p). We further tested two different degrees of freedom (*df*) for the hypothesized inverse Wishart distribution. Depending on the number of dimensions of the matrices being estimated, i.e. 11 for *Bromus* and 10 for *Trifolium*, *df* were represented by 0.01 or 10.01 for *Bromus* and 0.009 or 9.009 for *Trifolium*. Additionally, we explored parameter-expanded priors, with prior means set to zero and the diagonal of the prior covariance matrix set to 2500 (Tab. S2).

For each model, we ran three independent chains using a burn-in period of 160.000 followed by 960.000 MCMC steps and a thinning of 200, resulting in a final sample size for the posterior distribution of estimates of 4000. Convergence and mixing of the three chains were tested by visual inspection of trace plots, Gelman and Rubin's convergence diagnostics and autocorrelation tests (Gelman and Rubin 1992, Brooks and Gelman 1998, Hadfield 2019). As convergence was obtained in all cases, model outputs from all three chains were combined to obtain posterior distributions of all estimates.

For the results presented below we selected parameter-expanded priors, with df = 10.01 for *Bromus* and df = 9.009 for *Trifolium*, both with diagonal matrices of 2500. These prior specifications showed the best combination of a low deviance information criterion and a well mixing behaviour for both species (Tab. S3; see also all model fits in Tab. S4 – S11). In most cases, differences in priors and distributions had little effect on both, quantitative estimates and biological interpretations.

Table S1: Coordinates of seed collection locations for each natural source population of *B. erectus* and *T. pratense*.

Population	Species	Origin	Coordinates
1	*B. erectus*	Löbejün	51.632909, 11.921833
2	*B. erectus*	Liederstädt	51.319616, 11.603522
3	*B. erectus*	Wils	51.530159, 11.770463
4	*B. erectus*	Herrengosserstedt	51.155070, 11.482844
5	*B. erectus*	Ketzerbachtal	51.172496, 13.297917
1	*T. pratense*	Seeburg	51.501851, 11.703951
2	*T. pratense*	Friedersdorf	51.086623, 14.854392
3	*T. pratense*	Liederstädt	51.319616, 11.603522
4	*T. pratense*	Bennstedt	51.483374, 11.816481

Table S2: Summary of linear mixed effect model and generalized linear mixed effect model (*) analyses evaluating the effect of treatments (Control & Drought) on functional trait means. For each model, estimates for intercept (Int) and simulated 95% Credible Intervals (CI) are shown for the fixed effects, estimated standard deviations (SD) are given for the random effects (block, seed family - SF, seed family treatment interaction – SF:treat, residuals - resid). Traits with significant treatment effect are printed in bold, where higher and lower mean trait values are highlighted in blue and grey, respectively.

Species	Trait	Fixed effect				SD Random effects			
		Control		Drought		block	SF	SF: treat	resid
		Int	95% CI	Int	95% CI				
Bromus erectus	vegetative biomass (g)	**19.58**	(19.29, 19.87)	**14.75**	(14.46, 15.04)	0.22	0.82	0.19	1.69
	plant height (cm)	**76.51**	(74.01, 79.06)	**69.71**	(67.20, 72.21)	2.42	4.75	0.00	10.72
	leaf length (cm)	**27.94**	(26.10, 29.79)	**20.81**	(18.99, 22.66)	2.07	1.27	0.34	3.56
	leaf width (mm)	**3.90**	(3.79, 4.01)	**3.56**	(3.45, 3.68)	0.11	0.20	0.08	0.54
	C:N ratio	**29.86**	(29.03, 30.70)	**27.72**	(26.88, 28.56)	0.49	1.98	0.80	3.62
	SLA (mm^2/mg)	**13.26**	(12.69, 13.83)	**14.99**	(14.43, 15.56)	0.57	1.02	0.00	2.15
	hair density (n)*	**17.23**	(15.47, 19.27)	**21.82**	(19.50, 24.39)	0.08	0.36	0.22	-
	inflorescence length (cm)	**8.60**	(8.38, 8.81)	**9.11**	(8.90, 9.32)	0.13	0.35	0.42	1.58
	stem weight (g)	4.17	(4.12, 4.23)	4.15	(4.09, 4.20)	0.06	0.05	0.04	0.14
	start of flowering (d)	144.59	(144.07, 145.12)	144.58	(144.07, 145.12)	0.22	1.75	0.00	3.91
	inflorescences (n)*	11.34	(10.11, 12.77)	11.53	(10.24, 12.98)	0.03	0.46	0.33	-
Trifolium pratense	biomass (g)	**35.50**	(35.06, 35.95)	**17.08**	(16.62, 17.51)	0.25	0.85	1.18	3.10
	plant height (cm)	**46.56**	(45.91, 47.22)	**31.35**	(30.69, 32.01)	0.00	1.41	0.00	6.97
	plant width (cm)	**49.58**	(47.77, 51.37)	**27.01**	(25.25, 28.79)	1.41	3.86	2.52	11.00
	leaf length (mm)	**34.39**	(33.59, 35.16)	**28.40**	(27.61, 29.20)	0.56	2.53	0.00	4.25
	leaf width (mm)	**21.29**	(20.70, 21.88)	**16.62**	(16.03, 17.23)	0.51	1.47	0.39	2.91
	C:N ratio	**12.66**	(12.38, 12.94)	**12.30**	(12.01, 12.59)	0.25	0.32	0.28	1.18
	SLA (mm^2/mg)	**27.37**	(26.64, 28.10)	**23.37**	(22.64, 24.10)	0.59	1.18	0.87	5.03
	hair density	**3.90**	(3.74, 4.06)	**4.97**	(4.81, 5.13)	0.08	0.53	0.20	1.07
	start of flowering (d)	**165.58**	(164.61, 166.55)	**168.03**	(167.04, 169.03)	0.60	3.45	1.07	5.33
	inflorescences (n)*	**57.79**	(53.48, 62.38)	**16.10**	(14.84, 17.42)	0.06	0.24	0.19	-

Table S3: Summary of linear mixed effect model and generalized linear mixed effect model (*) analyses evaluating the effect of treatments (Control & Drought) on functional trait means including the partitioning of genetic variances at source population level. For each model, estimates for intercept (Int) and simulated 95% Credible Intervals (CI) are shown for the fixed effects, estimated standard deviations (SD) are given for the random effects (block, seed family - SF, seed family treatment interaction – SF:treat, natural source population – pop, natural source population treatment interaction – pop:treat, residuals - resid). Traits with significant treatment effect are printed in bold, where higher and lower mean trait values are highlighted in blue and grey, respectively. Results demonstrate that for the majority of trait and species combinations the among-population component is smaller than the within-population component.

**Species**	**Trait**	Fixed effect				SD Random effects					
		**Control**		**Drought**		block	SF	SF: treat	pop	pop: treat	resid.
		Int	95% CI	Int	95% CI						
Bromus erectus	vegetative biomass (g)	**19.58**	(19.28, 19.87)	**14.75**	(14.46, 15.05)	0.22	0.82	0.19	0.00	0.03	1.69
	plant height (cm)	**76.99**	(73.84, 80.04)	**70.19**	(67.12, 73.21)	2.42	4.55	0.00	1.95	0.00	10.72
	leaf length (cm)	**27.93**	(26.04, 29.83)	**20.82**	(18.95, 22.69)	2.07	1.27	0.34	0.00	0.00	3.56
	leaf width (mm)	**3.90**	(3.79, 4.01)	**3.56**	(3.45, 3.68)	0.11	0.20	0.08	0.02	0.00	0.54
	C:N ratio	**29.87**	(28.99, 30.74)	**27.72**	(26.86, 28.61)	0.49	1.96	0.80	0.26	0.10	3.62
	SLA (mm^2/mg)	**13.14**	(12.40, 13.89)	**14.89**	(14.15, 15.62)	0.57	0.95	0.00	0.46	0.29	2.15
	hair density (n)*	**17.44**	(14.94, 20.28)	**22.09**	(18.91, 25.74)	0.09	0.34	0.22	0.12	0.00	-
	inflorescence length (cm)	**8.64**	(8.32, 8.96)	**9.15**	(8.84, 9.47)	0.14	0.26	0.42	0.27	0.00	1.58
	stem weight (g)	4.17	(4.12, 4.23)	4.15	(4.09, 4.21)	0.06	0.05	0.04	0.00	0.00	0.14
	start of flowering (d)	144.50	(143.28, 145.71)	144.46	(143.24, 145.67)	0.23	1.29	0.00	1.21	0.27	3.91
	inflorescences (n)*	11.30	(9.63, 13.26)	11.48	(9.76, 13.45)	0.03	0.44	0.33	0.12	0.00	-
Trifolium pratense	biomass (g)	**35.51**	(34.69, 36.33)	**17.09**	(16.28, 17.91)	0.25	0.79	1.03	0.33	0.59	3.10
	plant height (cm)	**46.73**	(45.36, 48.10)	**31.39**	(30.05, 32.73)	0.00	1.10	0.00	0.74	0.93	6.94
	plant width (cm)	**50.69**	(46.45, 54.92)	**27.60**	(23.26, 31.74)	1.41	2.87	1.19	3.12	2.47	11.00
	leaf length (mm)	**34.19**	(32.13, 36.21)	**28.21**	(26.20, 30.22)	0.56	1.68	0.00	1.92	0.00	4.25
	leaf width (mm)	**21.12**	(19.86, 22.42)	**16.45**	(15.16, 17.72)	0.51	1.00	0.29	1.13	0.31	2.91
	C:N ratio	**12.66**	(12.34, 12.99)	**12.30**	(11.99, 12.62)	0.25	0.30	0.29	0.14	0.00	1.18
	SLA (mm^2/mg)	**27.46**	(26.39, 28.53)	**23.31**	(22.23, 24.39)	0.59	1.12	0.62	0.38	0.68	5.03
	hair density	**3.90**	(3.58, 4.23)	**4.98**	(4.65, 5.32)	0.08	0.47	0.17	0.26	0.13	1.07
	start of flowering (d)	165.57	(162.70, 168.40)	168.00	(165.20, 170.81)	0.60	2.64	1.04	2.70	0.30	5.33
	inflorescences (n)*	**59.26**	(48.65, 72.41)	**16.76**	(13.77, 20.38)	0.06	0.14	0.17	0.18	0.06	-

Table S4: Priors tested to best fit the model. We decided to use prior specification G11e2 and G10e2 for *Bromus* and *Trifolium*, respectively (coloured in blue) for genetic variance-covariance matrices. These models revealed to have the best combination of a low deviance information criterion (see Tab. S2) and well-behaved posterior distributions.

	**Prior specification**
Bromus erectus	
G11vp2:	list(R=list(V=V_P/2,nu=10.01),G=list(G1=list(V=V_P/2,nu=10.01)))
G11vp2.2:	list(R=list(V=V_P/2,nu=0.01),G=list(G1=list(V=V_P/2,nu=0.01)))
G11e2:	list(R=list(V=V_P/2,nu=10.01),G=list(G1=list(V=diag(11),nu=11,alpha.mu=c(rep(0,11)),alpha.V=diag(11)*2500)))
G11e3:	list(R=list(V=V_P/2,nu=0.010),G=list(G1=list(V=diag(11),nu=11,alpha.mu=c(rep(0,11)),alpha.V=diag(11)*2500)))
Trifolium pratense	
G10vp2:	list(R=list(V=V_P/2,nu=9.009),G=list(G1=list(V=V_P/2,nu=9.009)))
G10vp2.2:	list(R=list(V=V_P/2,nu=0.009), G=list(G1=list(V=V_P/2,nu=0.009)))
G10e2:	list(R=list(V=V_P/2,nu=9.009),G=list(G1=list(V=diag(10),nu=10,alpha.mu=c(rep(0,10)), alpha.V=diag(10)*2500)))
G10e3:	list(R=list(V=V_P/2,nu=0.009),G=list(G1=list(V=diag(10),nu=10,alpha.mu=c(rep(0,10)), alpha.V=diag(10)*2500)))

Table S5: Deviance Information Criteria (DIC) for all three independent chains computed for each model. For each species the model presented is coloured in blue.

Bromus erectus						
	**CON.1**	**CON.2**	**CON.3**	**DRY.1**	**DRY.2**	**DRY.3**
G11vp2:	DIC: 22118.16	DIC: 22118.26	DIC: 22118.72	DIC: 20965.89	DIC: 20965.54	DIC: 20965.60
G11vp2.2:	DIC: 22126.68	DIC: 22126.58	DIC: 22126.20	DIC: 20973.17	DIC: 20973.43	DIC: 20973.99
G11e2:	DIC: 22116.19	DIC: 22116.62	DIC: 22116.61	DIC: 20963.74	DIC: 20963.50	DIC: 20963.71
G11e3:	DIC: 22120.07	DIC: 22119.99	DIC: 22120.02	DIC: 20963.47	DIC: 20963.50	DIC: 20963.36
Trifolium pratense						
	**CON.1**	**CON.2**	**CON.3**	**DRY.1**	**DRY.2**	**DRY.3**
G10vp2:	DIC: 24360.77	DIC: 24360.50	DIC: 24360.70	DIC: 23023.90	DIC:23023.74	DIC: 23023.99
G10vp2.2:	DIC: 24365.54	DIC: 24365.83	DIC: 24365.87	DIC: 22948.42	DIC: 22948.48	DIC: 22948.58
G10e2:	DIC: 24339.78	DIC: 24340.09	DIC: 24339.81	DIC: 22947.58	DIC: 22947.79	DIC: 22947.86
G10e3:	DIC: 24338.93	DIC: 24339.16	DIC: 24339.08	DIC: 22947.00	DIC: 22946.85	DIC: 22946.51

Table S6: *Bromus erectus – G-Matrix of prior specification G11e2:* Posterior mean MCMC estimates of G for both treatments (CON & DRY), with 95% Bayesian Credible intervals. Traits with significant treatment effect in genetic variances coloured in yellow; significant genetic covariances between trait pairs except with relative fitness coloured in green; significant genetic covariances with relative fitness in blue.

G11e2	biomass	leaf length	leaf width	SLA	hair density	plant height	inflo length	stem weight	flowering time	C:N ratio
**biomass**	CON: 0.87<br>(0.39, 1.48)<br>DRY: 0.57<br>(0.36, 0.86)									
**leaf length**	CON: 0.20<br>(-0.31, 0.75)<br>DRY: -0.02<br>(-0.28, 0.25)	CON: 2.15<br>(0.76, 3.98)<br>DRY: 0.80<br>(0.03, 1.87)								
**leaf width**	CON: -0.02<br>(-0.11, 0.08)<br>DRY: -0.02<br>(-0.07, 0.02)	CON: -0.16<br>(-0.32, -0.02)<br>DRY: 0.00<br>(-0.07, 0.07)	CON: 0.07<br>(0.03, 0.11)<br>DRY: 0.02<br>(0.00, 0.05)							
**SLA**	CON: -0.34<br>(-0.77, 0.04)<br>DRY: -0.03<br>(-0.24, 0.16)	CON: -0.32<br>(-1.07, 0.29)<br>DRY: -0.09<br>(-0.46, 0.19)	CON: 0.05<br>(-0.05, 0.17)<br>DRY: 0.00<br>(-0.05, 0.05)	CON: 1.46<br>(0.79, 2.33)<br>DRY: 0.57<br>(0.17, 1.05)						
**hair density**	CON: -0.69<br>(-2.95, 1.45)<br>DRY: 0.82<br>(-0.79, 2.58)	CON: -0.63<br>(-4.26, 2.91)<br>DRY: 1.34<br>(-1.32, 4.33)	CON: 0.03<br>(-0.57, 0.63)<br>DRY: -0.39<br>(-0.89, 0.03)	CON: -2.22<br>(-5.00, 0.31)<br>DRY: -1.15<br>(-3.37, 0.81)	CON: 53.91<br>(34.25, 78.73)<br>DRY: 66.28<br>(43.14, 96.14)					
**plant height**	CON: 0.60<br>(-0.61, 1.97)<br>DRY: -0.49<br>(-1.62, 0.60)	CON: 1.64<br>(-0.30, 4.14)<br>DRY: 0.83<br>(-0.79, 3.19)	CON: -0.09<br>(-0.45, 0.27)<br>DRY: 0.21<br>(-0.05, 0.57)	CON: -1.02<br>(-2.78, 0.41)<br>DRY: -0.30<br>(-1.78, 0.97)	CON: 0.61<br>(-7.83, 9.18)<br>DRY: -3.38<br>(-15.01, 7.52)	CON: 9.62<br>(1.45, 19.66)<br>DRY: 15.01<br>(2.78, 31.02)				
**inflo length**	CON: -0.01<br>(-0.16, 0.10)<br>DRY: 0.02<br>(-0.12, 0.16)	CON: 0.05<br>(-0.13, 0.33)<br>DRY: 0.07<br>(-0.14, 0.34)	CON: 0.00<br>(-0.05, 0.03)<br>DRY: 0.01<br>(-0.03, 0.05)	CON: 0.00<br>(-0.16, 0.17)<br>DRY: 0.07<br>(-0.09, 0.26)	CON: -0.25<br>(-1.34, 0.47)<br>DRY: 0.26<br>(-1.22, 1.83)	CON: 0.10<br>(-0.27, 0.77)<br>DRY: 0.54<br>(-0.41, 2.05)	CON: 0.06<br>(0.00, 0.23)<br>DRY: 0.24<br>(0.01, 0.55)			
**stem weight**	CON: -0.01<br>(-0.02, 0.01)<br>DRY: -0.01<br>(-0.02, 0.01)	CON: -0.01<br>(-0.04, 0.02)<br>DRY: 0.01<br>(-0.01, 0.04)	CON: 0.01<br>(0.00, 0.01)<br>DRY: 0.00<br>(0.00, 0.01)	CON: 0.00<br>(-0.02, 0.02)<br>DRY: 0.00<br>(-0.02, 0.02)	CON: 0.04<br>(-0.08, 0.16)<br>DRY: 0.03<br>(-0.12, 0.19)	CON: 0.02<br>(-0.04, 0.12)<br>DRY: 0.14<br>(0.01, 0.32)	CON: 0.00<br>(0.00, 0.01)<br>DRY: 0.01<br>(0.00, 0.03)	CON: 0.00<br>(0.00, 0.00)<br>DRY: 0.00<br>(0.00, 0.01)		
**flowering time**	CON: -0.09<br>(-0.68, 0.47)<br>DRY: 0.43<br>(0.00, 0.91)	CON: 0.47<br>(-0.49, 1.48)<br>DRY: 0.05<br>(-0.66, 0.76)	CON: -0.16<br>(-0.34, 0.00)<br>DRY: -0.08<br>(-0.21, 0.02)	CON: -0.57<br>(-1.34, 0.12)<br>DRY: 0.16<br>(-0.36, 0.70)	CON: 2.09<br>(-1.62, 6.11)<br>DRY: 2.56<br>(-1.94, 7.39)	CON: 0.19<br>(-2.14, 2.54)<br>DRY: -1.90<br>(-5.26, 0.88)	CON: 0.01<br>(-0.23, 0.26)<br>DRY: 0.04<br>(-0.36, 0.43)	CON: -0.02<br>(-0.06, 0.01)<br>DRY: -0.01<br>(-0.05, 0.03)	CON: 2.90<br>(1.39, 4.87)<br>DRY: 3.01<br>(1.09, 5.51)	
**C:N ratio**	CON: 0.79<br>(-0.08, 1.82)<br>DRY: -0.02<br>(-0.46, 0.46)	CON: 0.07<br>(-1.43, 1.60)<br>DRY: 0.21<br>(-0.46, 0.98)	CON: 0.01<br>(-0.25, 0.28)<br>DRY: 0.06<br>(-0.05, 0.19)	CON: -0.74<br>(-1.98, 0.35)<br>DRY: -0.02<br>(-0.59, 0.47)	CON: 0.99<br>(-5.53, 7.48)<br>DRY: -1.23<br>(-6.24, 3.57)	CON: 0.51<br>(-3.04, 4.14)<br>DRY: 2.55<br>(-0.46, 6.27)	CON: -0.03<br>(-0.44, 0.31)<br>DRY: 0.12<br>(-0.27, 0.56)	CON: 0.02<br>(-0.04, 0.07)<br>DRY: 0.05<br>(0.00, 0.10)	CON: -1.00<br>(-2.87, 0.73)<br>DRY: -0.18<br>(-1.40, 1.05)	CON: 5.92<br>(2.73, 10.25)<br>DRY: 2.34<br>(0.69, 4.66)
**Relative fitness**	CON: -0.04<br>(-0.17, 0.08)<br>DRY: -0.02<br>(-0.10, 0.07)	CON: 0.00<br>(-0.22, 0.22)<br>DRY: 0.03<br>(-0.11, 0.17)	CON: -0.02<br>(-0.06, 0.02)<br>DRY: -0.01<br>(-0.03, 0.01)	CON: -0.06<br>(-0.23, 0.09)<br>DRY: -0.02<br>(-0.13, 0.08)	CON: 0.46<br>(-0.38, 1.34)<br>DRY: 0.64<br>(-0.21, 1.56)	CON: -0.15<br>(-0.68, 0.34)<br>DRY: -0.47<br>(-1.14, 0.09)	CON: 0.00<br>(-0.06, 0.05)<br>DRY: 0.01<br>(-0.07, 0.09)	CON: 0.00<br>(-0.01, 0.00)<br>DRY: -0.01<br>(-0.01, 0.00)	CON: 0.34<br>(0.12, 0.61)<br>DRY: 0.07<br>(-0.15, 0.31)	CON: -0.27<br>(-0.68, 0.10)<br>DRY: -0.23<br>(-0.51, -0.01)

Table S7: *Bromus erectus – G-Matrix of prior specification G11vp2:* Posterior mean MCMC estimates of G for both treatments (CON & DRY), with 95% Bayesian Credible intervals. Traits with significant treatment effect in genetic variances coloured in yellow; significant genetic covariances between trait pairs except with relative fitness coloured in green; significant genetic covariances with relative fitness in blue.

G11vp2	biomass	leaf length	leaf width	SLA	hair density	plant height	inflo length	stem weight	flowering time	C:N ratio
**biomass**	CON: 1.47<br>(0.99, 2.10)<br>DRY: 0.71<br>(0.49, 1.00)									
**leaf length**	CON: 0.18<br>(-0.52, 0.91)<br>DRY: 0.05<br>(-0.33, 0.45)	CON: 4.88<br>(3.32, 7.03)<br>DRY: 3.03<br>(2.08, 4.32)								
**leaf width**	CON: -0.01<br>(-0.12, 0.11)<br>DRY: -0.02<br>(-0.08, 0.04)	CON: -0.12<br>(-0.33, 0.08)<br>DRY: 0.01<br>(-0.11, 0.13)	CON: 0.12<br>(0.08, 0.17)<br>DRY: 0.07<br>(0.05, 0.10)							
**SLA**	CON: -0.35<br>(-0.86, 0.11)<br>DRY: -0.09<br>(-0.35, 0.15)	CON: -0.58<br>(-1.53, 0.25)<br>DRY: -0.27<br>(-0.82, 0.22)	CON: 0.05<br>(-0.08, 0.19)<br>DRY: 0.00<br>(-0.08, 0.07)	CON: 2.16<br>(1.47, 3.07)<br>DRY: 1.17<br>(0.79, 1.68)						
**hair density**	CON: -0.69<br>(-3.32, 1.81)<br>DRY: 0.81<br>(-1.02, 2.76)	CON: -0.81<br>(-5.65, 3.74)<br>DRY: 1.05<br>(-2.94, 5.09)	CON: 0.01<br>(-0.70, 0.72)<br>DRY: -0.39<br>(-1.02, 0.20)	CON: -2.06<br>(-5.25, 0.89)<br>DRY: -1.23<br>(-3.84, 1.19)	CON: 62.84<br>(43.88, 88.30)<br>DRY: 75.59<br>(52.48, 105.70)					
**plant height**	CON: 0.50<br>(-1.37, 2.48)<br>DRY: -0.26<br>(-1.75, 1.22)	CON: 2.81<br>(-0.43, 6.52)<br>DRY: 2.26<br>(-0.70, 5.74)	CON: 0.02<br>(-0.51, 0.55)<br>DRY: 0.32<br>(-0.13, 0.84)	CON: -1.43<br>(-3.91, 0.80)<br>DRY: -0.75<br>(-2.78, 1.13)	CON: -0.66<br>(-12.98, 11.39)<br>DRY: -2.78<br>(-18.39, 12.28)	CON: 31.30<br>(20.71, 45.86)<br>DRY: 41.80<br>(27.73, 60.80)				
**inflo length**	CON: -0.04<br>(-0.30, 0.23)<br>DRY: 0.02<br>(-0.18, 0.22)	CON: 0.26<br>(-0.19, 0.77)<br>DRY: 0.15<br>(-0.26, 0.58)	CON: 0.00<br>(-0.07, 0.08)<br>DRY: 0.02<br>(-0.04, 0.09)	CON: -0.05<br>(-0.37, 0.27)<br>DRY: 0.06<br>(-0.20, 0.33)	CON: -0.64<br>(-2.40, 1.06)<br>DRY: 0.42<br>(-1.60, 2.56)	CON: 1.19<br>(0.02, 2.67)<br>DRY: 1.68<br>(0.13, 3.56)	CON: 0.59<br>(0.39, 0.86)<br>DRY: 0.76<br>(0.50, 1.11)			
**stem weight**	CON: -0.01<br>(-0.04, 0.02)<br>DRY: 0.00<br>(-0.02, 0.01)	CON: 0.01<br>(-0.04, 0.06)<br>DRY: 0.01<br>(-0.03, 0.05)	CON: 0.01<br>(0.00, 0.02)<br>DRY: 0.00<br>(0.00, 0.01)	CON: -0.01<br>(-0.04, 0.02)<br>DRY: 0.00<br>(-0.03, 0.02)	CON: 0.01<br>(-0.17, 0.19)<br>DRY: 0.05<br>(-0.15, 0.25)	CON: 0.16<br>(0.04, 0.32)<br>DRY: 0.27<br>(0.11, 0.46)	CON: 0.02<br>(0.00, 0.04)<br>DRY: 0.03<br>(0.01, 0.05)	CON: 0.01<br>(0.00, 0.01)<br>DRY: 0.01<br>(0.00, 0.01)		
**flowering time**	CON: -0.12<br>(-0.88, 0.60)<br>DRY: 0.41<br>(-0.12, 0.99)	CON: 0.43<br>(-0.88, 1.77)<br>DRY: -0.08<br>(-1.25, 1.04)	CON: -0.15<br>(-0.36, 0.05)<br>DRY: -0.10<br>(-0.28, 0.07)	CON: -0.56<br>(-1.49, 0.27)<br>DRY: 0.10<br>(-0.61, 0.84)	CON: 1.78<br>(-2.87, 6.50)<br>DRY: 2.12<br>(-3.28, 7.88)	CON: 0.08<br>(-3.38, 3.56)<br>DRY: -2.53<br>(-7.24, 1.77)	CON: 0.01<br>(-0.48, 0.49)<br>DRY: -0.04<br>(-0.64, 0.54)	CON: -0.02<br>(-0.07, 0.03)<br>DRY: -0.02<br>(-0.07, 0.04)	CON: 4.91<br>(3.30, 7.06)<br>DRY: 5.77<br>(3.85, 8.40)	
**C:N ratio**	CON: 0.89<br>(-0.14, 2.09)<br>DRY: 0.14<br>(-0.44, 0.76)	CON: 0.00<br>(-1.94, 1.97)<br>DRY: 0.28<br>(-0.86, 1.45)	CON: 0.01<br>(-0.30, 0.32)<br>DRY: 0.05<br>(-0.12, 0.24)	CON: -0.72<br>(-2.14, 0.55)<br>DRY: -0.20<br>(-0.96, 0.52)	CON: 0.97<br>(-6.26, 8.47)<br>DRY: -1.24<br>(-7.57, 4.80)	CON: -0.03<br>(-5.37, 5.23)<br>DRY: 2.49<br>(-1.92, 7.27)	CON: -0.12<br>(-0.90, 0.61)<br>DRY: 0.07<br>(-0.53, 0.67)	CON: 0.01<br>(-0.07, 0.09)<br>DRY: 0.04<br>(-0.02, 0.11)	CON: -0.97<br>(-3.14, 1.09)<br>DRY: -0.11<br>(-1.79, 1.59)	CON: 8.52<br>(5.28, 13.25)<br>DRY: 4.90<br>(3.03, 7.62)
**Relative fitness**	CON: -0.04<br>(-0.20, 0.11)<br>DRY: -0.02<br>(-0.12, 0.08)	CON: -0.01<br>(-0.29, 0.28)<br>DRY: 0.03<br>(-0.16, 0.23)	CON: -0.02<br>(-0.06, 0.03)<br>DRY: -0.01<br>(-0.04, 0.02)	CON: -0.06<br>(-0.25, 0.12)<br>DRY: -0.03<br>(-0.16, 0.10)	CON: 0.40<br>(-0.57, 1.40)<br>DRY: 0.56<br>(-0.38, 1.57)	CON: -0.14<br>(-0.87, 0.61)<br>DRY: -0.49<br>(-1.31, 0.24)	CON: 0.00<br>(-0.10, 0.11)<br>DRY: 0.02<br>(-0.09, 0.13)	CON: -0.01<br>(-0.02, 0.01)<br>DRY: -0.01<br>(-0.02, 0.00)	CON: 0.33<br>(0.05, 0.65)<br>DRY: 0.07<br>(-0.21, 0.35)	CON: -0.25<br>(-0.71, 0.18)<br>DRY: -0.25<br>(-0.58, 0.03)

Table S8: *Bromus erectus – G-Matrix of prior specification G11vp2.2:* Posterior mean MCMC estimates of G for both treatments (CON & DRY), with 95% Bayesian Credible intervals. Traits with significant treatment effect in genetic variances coloured in yellow; significant genetic covariances between trait pairs except with relative fitness coloured in green; significant genetic covariances with relative fitness in blue.

G11vp2.2	biomass	leaf length	leaf width	SLA	hair density	plant height	inflo length	stem weight	flowering time	C:N ratio
**biomass**	CON: 0.87<br>(0.39, 1.49)<br>DRY: 0.57<br>(0.34, 0.86)									
**leaf length**	CON: 0.21<br>(-0.45, 0.91)<br>DRY: -0.06<br>(-0.42, 0.30)	CON: 2.36<br>(1.01, 4.22)<br>DRY: 0.94<br>(0.30, 1.91)								
**leaf width**	CON: -0.03<br>(-0.14, 0.08)<br>DRY: -0.03<br>(-0.09, 0.02)	CON: -0.22<br>(-0.42, -0.05)<br>DRY: 0.00<br>(-0.09, 0.09)	CON: 0.07<br>(0.03, 0.12)<br>DRY: 0.02<br>(0.01, 0.05)							
**SLA**	CON: -0.48<br>(-1.00, -0.02)<br>DRY: -0.04<br>(-0.30, 0.20)	CON: -0.41<br>(-1.33, 0.37)<br>DRY: -0.14<br>(-0.62, 0.27)	CON: 0.06<br>(-0.07, 0.20)<br>DRY: 0.00<br>(-0.07, 0.07)	CON: 1.52<br>(0.83, 2.43)<br>DRY: 0.54<br>(0.19, 1.02)						
**hair density**	CON: -1.03<br>(-3.60, 1.45)<br>DRY: 1.01<br>(-0.88, 3.03)	CON: -1.00<br>(-5.50, 3.39)<br>DRY: 2.44<br>(-1.16, 6.20)	CON: 0.02<br>(-0.68, 0.74)<br>DRY: -0.63<br>(-1.23, -0.09)	CON: -2.84<br>(-6.24, 0.20)<br>DRY: -1.84<br>(-4.49, 0.51)	CON: 53.89<br>(33.88, 79.71)<br>DRY: 66.16<br>(42.01, 97.25)					
**plant height**	CON: 0.62<br>(-1.23, 2.56)<br>DRY: -0.68<br>(-2.16, 0.79)	CON: 3.14<br>(0.17, 6.82)<br>DRY: 1.76<br>(-0.75, 4.87)	CON: -0.07<br>(-0.56, 0.45)<br>DRY: 0.36<br>(-0.02, 0.83)	CON: -1.88<br>(-4.29, 0.28)<br>DRY: -0.57<br>(-2.59, 1.20)	CON: -0.30<br>(-12.45, 11.93)<br>DRY: -2.51<br>(-17.44, 12.46)	CON: 14.33<br>(5.06, 27.66)<br>DRY: 21.68<br>(8.06, 40.42)				
**inflo length**	CON: -0.08<br>(-0.33, 0.17)<br>DRY: 0.03<br>(-0.17, 0.23)	CON: 0.21<br>(-0.16, 0.65)<br>DRY: 0.16<br>(-0.16, 0.53)	CON: -0.01<br>(-0.07, 0.06)<br>DRY: 0.02<br>(-0.03, 0.08)	CON: -0.04<br>(-0.35, 0.25)<br>DRY: 0.12<br>(-0.12, 0.36)	CON: -0.90<br>(-2.62, 0.68)<br>DRY: 0.42<br>(-1.54, 2.52)	CON: 0.71<br>(-0.26, 2.06)<br>DRY: 1.24<br>(-0.21, 3.15)	CON: 0.18<br>(0.04, 0.40)<br>DRY: 0.33<br>(0.11, 0.66)			
**stem weight**	CON: -0.01<br>(-0.04, 0.01)<br>DRY: -0.01<br>(-0.03, 0.01)	CON: -0.01<br>(-0.05, 0.04)<br>DRY: 0.02<br>(-0.01, 0.06)	CON: 0.01<br>(0.00, 0.02)<br>DRY: 0.00<br>(0.00, 0.01)	CON: -0.01<br>(-0.04, 0.02)<br>DRY: 0.00<br>(-0.03, 0.02)	CON: 0.04<br>(-0.14, 0.22)<br>DRY: 0.06<br>(-0.13, 0.27)	CON: 0.09<br>(-0.02, 0.25)<br>DRY: 0.25<br>(0.09, 0.47)	CON: 0.01<br>(0.00, 0.03)<br>DRY: 0.02<br>(0.00, 0.05)	CON: 0.00<br>(0.00, 0.01)<br>DRY: 0.00<br>(0.00, 0.01)		
**flowering time**	CON: -0.15<br>(-0.87, 0.53)<br>DRY: 0.62<br>(0.11, 1.20)	CON: 0.60<br>(-0.55, 1.85)<br>DRY: 0.07<br>(-0.84, 0.97)	CON: -0.21<br>(-0.42, -0.02)<br>DRY: -0.12<br>(-0.27, 0.01)	CON: -0.77<br>(-1.68, 0.04)<br>DRY: 0.29<br>(-0.35, 0.95)	CON: 2.90<br>(-1.58, 7.66)<br>DRY: 3.34<br>(-1.96, 9.01)	CON: 0.23<br>(-2.98, 3.41)<br>DRY: -2.72<br>(-7.07, 1.14)	CON: 0.02<br>(-0.39, 0.45)<br>DRY: 0.07<br>(-0.46, 0.58)	CON: -0.03<br>(-0.08, 0.02)<br>DRY: -0.01<br>(-0.07, 0.04)	CON: 2.87<br>(1.37, 4.90)<br>DRY: 2.69<br>(1.07, 4.99)	
**C:N ratio**	CON: 1.14<br>(0.02, 2.42)<br>DRY: 0.00<br>(-0.58, 0.63)	CON: 0.05<br>(-1.96, 2.01)<br>DRY: 0.37<br>(-0.56, 1.40)	CON: -0.01<br>(-0.32, 0.32)<br>DRY: 0.10<br>(-0.05, 0.26)	CON: -1.03<br>(-2.52, 0.29)<br>DRY: 0.01<br>(-0.74, 0.68)	CON: 2.02<br>(-5.81, 10.11)<br>DRY: -2.10<br>(-8.62, 4.12)	CON: 0.25<br>(-5.50, 5.91)<br>DRY: 4.42<br>(0.28, 9.26)	CON: -0.13<br>(-0.96, 0.64)<br>DRY: 0.25<br>(-0.32, 0.85)	CON: 0.02<br>(-0.07, 0.11)<br>DRY: 0.08<br>(0.02, 0.14)	CON: -1.53<br>(-3.87, 0.55)<br>DRY: -0.12<br>(-1.65, 1.48)	CON: 6.31<br>(2.84, 11.04)<br>DRY: 2.81<br>(1.09, 5.29)
**Relative fitness**	CON: -0.06<br>(-0.22, 0.10)<br>DRY: -0.03<br>(-0.13, 0.06)	CON: -0.03<br>(-0.30, 0.23)<br>DRY: 0.05<br>(-0.14, 0.25)	CON: -0.03<br>(-0.07, 0.02)<br>DRY: -0.02<br>(-0.04, 0.01)	CON: -0.07<br>(-0.26, 0.11)<br>DRY: -0.02<br>(-0.15, 0.10)	CON: 0.59<br>(-0.40, 1.62)<br>DRY: 0.76<br>(-0.20, 1.81)	CON: -0.39<br>(-1.11, 0.34)<br>DRY: -0.70<br>(-1.54, 0.05)	CON: -0.01<br>(-0.11, 0.08)<br>DRY: 0.01<br>(-0.09, 0.12)	CON: -0.01<br>(-0.02, 0.00)<br>DRY: -0.01<br>(-0.02, 0.00)	CON: 0.49<br>(0.22, 0.81)<br>DRY: 0.08<br>(-0.19, 0.37)	CON: -0.43<br>(-0.94, 0.03)<br>DRY: -0.37<br>(-0.72, -0.08)

Table S9: *Bromus erectus – G-Matrix of prior specification G11e3:* Posterior mean MCMC estimates of G for both treatments (CON & DRY), with 95% Bayesian Credible intervals. Traits with significant treatment effect in genetic variances coloured in yellow; significant genetic covariances between trait pairs except with relative fitness coloured in green; significant genetic covariances with relative fitness in blue.

G11e3	biomass	leaf length	leaf width	SLA	hair density	plant height	inflo length	stem weight	flowering time	C:N ratio
biomass	CON: 0.86<br>(0.38, 1.47)<br>DRY: 0.57<br>(0.36, 0.86)									
leaf length	CON: 0.20<br>(-0.33, 0.75)<br>DRY: -0.02<br>(-0.28, 0.25)	CON: 2.10<br>(0.71, 3.88)<br>DRY: 0.80<br>(0.03, 1.86)								
leaf width	CON: -0.02<br>(-0.11, 0.07)<br>DRY: -0.02<br>(-0.07, 0.02)	CON: -0.16<br>(-0.31, -0.02)<br>DRY: 0.00<br>(-0.07, 0.06)	CON: 0.07<br>(0.03, 0.11)<br>DRY: 0.02<br>(0.00, 0.05)							
SLA	CON: -0.33<br>(-0.77, 0.04)<br>DRY: -0.03<br>(-0.24, 0.15)	CON: -0.30<br>(-1.04, 0.31)<br>DRY: -0.08<br>(-0.45, 0.20)	CON: 0.05<br>(-0.05, 0.17)<br>DRY: 0.00<br>(-0.05, 0.05)	CON: 1.45<br>(0.80, 2.29)<br>DRY: 0.56<br>(0.17, 1.05)						
hair density	CON: -0.70<br>(-2.94, 1.45)<br>DRY: 0.81<br>(-0.85, 2.56)	CON: -0.66<br>(-4.33, 2.81)<br>DRY: 1.31<br>(-1.36, 4.29)	CON: 0.02<br>(-0.57, 0.63)<br>DRY: -0.38<br>(-0.89, 0.04)	CON: -2.20<br>(-5.01, 0.37)<br>DRY: -1.13<br>(-3.30, 0.85)	CON: 53.75<br>(34.02, 79.54)<br>DRY: 66.17<br>(42.99, 96.18)					
plant height	CON: 0.61<br>(-0.61, 2.00)<br>DRY: -0.49<br>(-1.59, 0.59)	CON: 1.58<br>(-0.33, 4.09)<br>DRY: 0.82<br>(-0.78, 3.19)	CON: -0.09<br>(-0.45, 0.27)<br>DRY: 0.21<br>(-0.05, 0.57)	CON: -1.01<br>(-2.79, 0.46)<br>DRY: -0.31<br>(-1.80, 0.94)	CON: 0.61<br>(-7.89, 9.04)<br>DRY: -3.31<br>(-15.17, 8.10)	CON: 9.65<br>(1.47, 20.25)<br>DRY: 15.05<br>(2.91, 30.65)				
inflo length	CON: -0.01<br>(-0.16, 0.10)<br>DRY: 0.02<br>(-0.13, 0.16)	CON: 0.05<br>(-0.12, 0.32)<br>DRY: 0.07<br>(-0.14, 0.34)	CON: 0.00<br>(-0.05, 0.03)<br>DRY: 0.01<br>(-0.03, 0.05)	CON: 0.00<br>(-0.16, 0.16)<br>DRY: 0.07<br>(-0.09, 0.26)	CON: -0.25<br>(-1.34, 0.48)<br>DRY: 0.26<br>(-1.22, 1.84)	CON: 0.10<br>(-0.27, 0.76)<br>DRY: 0.54<br>(-0.39, 1.99)	CON: 0.06<br>(0.00, 0.24)<br>DRY: 0.24<br>(0.01, 0.55)			
stem weight	CON: 0.00<br>(-0.02, 0.01)<br>DRY: -0.01<br>(-0.02, 0.01)	CON: -0.01<br>(-0.04, 0.02)<br>DRY: 0.01<br>(-0.01, 0.04)	CON: 0.01<br>(0.00, 0.01)<br>DRY: 0.00<br>(0.00, 0.01)	CON: 0.00<br>(-0.02, 0.02)<br>DRY: 0.00<br>(-0.02, 0.02)	CON: 0.04<br>(-0.08, 0.16)<br>DRY: 0.03<br>(-0.12, 0.19)	CON: 0.02<br>(-0.04, 0.12)<br>DRY: 0.14<br>(0.01, 0.32)	CON: 0.00<br>(0.00, 0.01)<br>DRY: 0.01<br>(0.00, 0.03)	CON: 0.00<br>(0.00, 0.00)<br>DRY: 0.00<br>(0.00, 0.01)		
flowering time	CON: -0.09<br>(-0.68, 0.47)<br>DRY: 0.43<br>(0.00, 0.92)	CON: 0.46<br>(-0.46, 1.48)<br>DRY: 0.05<br>(-0.66, 0.77)	CON: -0.16<br>(-0.34, 0.00)<br>DRY: -0.08<br>(-0.21, 0.02)	CON: -0.57<br>(-1.33, 0.11)<br>DRY: 0.17<br>(-0.35, 0.72)	CON: 2.07<br>(-1.79, 6.12)<br>DRY: 2.56<br>(-2.03, 7.49)	CON: 0.19<br>(-2.12, 2.52)<br>DRY: -1.89<br>(-5.22, 0.82)	CON: 0.01<br>(-0.23, 0.25)<br>DRY: 0.04<br>(-0.34, 0.43)	CON: -0.02<br>(-0.06, 0.01)<br>DRY: -0.01<br>(-0.05, 0.03)	CON: 2.85<br>(1.32, 4.83)<br>DRY: 3.01<br>(1.07, 5.48)	
C:N ratio	CON: 0.76<br>(-0.10, 1.79)<br>DRY: -0.01<br>(-0.44, 0.48)	CON: 0.06<br>(-1.42, 1.57)<br>DRY: 0.20<br>(-0.47, 0.97)	CON: 0.01<br>(-0.24, 0.28)<br>DRY: 0.06<br>(-0.05, 0.19)	CON: -0.72<br>(-1.92, 0.38)<br>DRY: -0.03<br>(-0.58, 0.50)	CON: 0.95<br>(-5.48, 7.27)<br>DRY: -1.24<br>(-6.27, 3.55)	CON: 0.49<br>(-3.05, 4.09)<br>DRY: 2.56<br>(-0.41, 6.27)	CON: -0.03<br>(-0.42, 0.31)<br>DRY: 0.11<br>(-0.29, 0.55)	CON: 0.02<br>(-0.03, 0.07)<br>DRY: 0.05<br>(0.00, 0.10)	CON: -0.95<br>(-2.89, 0.72)<br>DRY: -0.19<br>(-1.42, 1.05)	CON: 5.78<br>(2.68, 10.11)<br>DRY: 2.37<br>(0.69, 4.70)
Relative fitness	CON: -0.04<br>(-0.17, 0.09)<br>DRY: -0.02<br>(-0.10, 0.07)	CON: 0.00<br>(-0.21, 0.22)<br>DRY: 0.03<br>(-0.11, 0.17)	CON: -0.02<br>(-0.06, 0.02)<br>DRY: -0.01<br>(-0.04, 0.01)	CON: -0.06<br>(-0.22, 0.09)<br>DRY: -0.02<br>(-0.13, 0.08)	CON: 0.46<br>(-0.37, 1.34)<br>DRY: 0.63<br>(-0.20, 1.52)	CON: -0.15<br>(-0.69, 0.35)<br>DRY: -0.47<br>(-1.12, 0.07)	CON: 0.00<br>(-0.05, 0.06)<br>DRY: 0.01<br>(-0.07, 0.09)	CON: 0.00<br>(-0.01, 0.00)<br>DRY: -0.01<br>(-0.01, 0.00)	CON: 0.34<br>(0.11, 0.61)<br>DRY: 0.08<br>(-0.15, 0.32)	CON: -0.27<br>(-0.69, 0.09)<br>DRY: -0.23<br>(-0.51, -0.01)

Table S10: *Trifolium pratense – G-Matrix of prior Specification G10e2:* Posterior mean MCMC estimates of G for both treatments (CON & DRY), with 95% Bayesian Credible intervals in brackets. Traits with significant treatment effect in genetic variances coloured in yellow; significant genetic covariances between trait pairs except with relative fitness coloured in green; significant genetic covariances with relative fitness coloured in blue.

G10e2	biomass	leaf length	leaf width	SLA	hair density	plant height	plant width	flowering time	C:N ratio
**biomass**	CON: 3.09<br>(1.52, 5.10)<br>DRY: 0.48<br>(0.17, 0.87)								
**leaf length**	CON: 2.69<br>(1.19, 4.54)<br>DRY: 0.29<br>(-0.34, 1.01)	CON: 5.72<br>(3.26, 8.90)<br>DRY: 5.73<br>(3.54, 8.57)							
**leaf width**	CON: 1.60<br>(0.65, 2.79)<br>DRY: 0.07<br>(-0.33, 0.51)	CON: 2.73<br>(1.37, 4.52)<br>DRY: 3.01<br>(1.72, 4.67)	CON: 2.08<br>(1.06, 3.39)<br>DRY: 2.22<br>(1.30, 3.40)						
**SLA**	CON: 0.41<br>(-0.74, 1.64)<br>DRY: 0.02<br>(-0.33, 0.40)	CON: 0.63<br>(-0.93, 2.31)<br>DRY: 1.86<br>(0.58, 3.40)	CON: 0.50<br>(-0.42, 1.60)<br>DRY: 1.23<br>(0.40, 2.26)	CON: 2.25<br>(0.09, 5.11)<br>DRY: 1.30<br>(0.29, 2.77)					
**hair density**	CON: -0.14<br>(-0.46, 0.17)<br>DRY: 0.01<br>(-0.16, 0.17)	CON: -0.50<br>(-0.99, -0.11)<br>DRY: -0.79<br>(-1.37, -0.33)	CON: -0.35<br>(-0.67, -0.09)<br>DRY: -0.46<br>(-0.84, -0.17)	CON: -0.02<br>(-0.35, 0.29)<br>DRY: -0.28<br>(-0.64, 0.00)	CON: 0.24<br>(0.13, 0.39)<br>DRY: 0.37<br>(0.21, 0.58)				
**plant height**	CON: 0.31<br>(-0.57, 1.62)<br>DRY: 0.12<br>(-0.22, 0.63)	CON: 0.54<br>(-0.82, 2.38)<br>DRY: 1.70<br>(0.03, 3.75)	CON: 0.26<br>(-0.60, 1.33)<br>DRY: 0.96<br>(-0.03, 2.24)	CON: 0.12<br>(-0.51, 1.07)<br>DRY: 0.67<br>(-0.06, 1.91)	CON: -0.06<br>(-0.37, 0.16)<br>DRY: -0.31<br>(-0.76, 0.01)	CON: 0.72<br>(0.00, 3.12)<br>DRY: 1.24<br>(0.03, 3.38)			
**plant width**	CON: -5.50<br>(-9.70, -1.96)<br>DRY: 0.00<br>(-0.56, 0.66)	CON: -10.02<br>(-16.12, -5.11)<br>DRY: -3.70<br>(-6.16, -1.71)	CON: -5.96<br>(-9.80, -2.87)<br>DRY: -2.41<br>(-3.91, -1.16)	CON: -1.52<br>(-5.31, 2.17)<br>DRY: -1.49<br>(-2.70, -0.49)	CON: 0.85<br>(-0.11, 1.93)<br>DRY: 0.60<br>(0.17, 1.13)	CON: -0.44<br>(-3.65, 3.42)<br>DRY: -1.00<br>(-2.25, 0.22)	CON: 34.84<br>(18.77, 55.54)<br>DRY: 4.01<br>(1.37, 7.70)		
**flowering time**	CON: 0.30<br>(-1.39, 2.05)<br>DRY: 0.43<br>(-0.56, 1.44)	CON: -0.78<br>(-3.05, 1.43)<br>DRY: 3.60<br>(0.78, 6.77)	CON: -1.01<br>(-2.52, 0.36)<br>DRY: 1.45<br>(-0.35, 3.37)	CON: 0.07<br>(-1.75, 1.87)<br>DRY: 1.82<br>(0.17, 3.79)	CON: 0.77<br>(0.31, 1.30)<br>DRY: -0.18<br>(-0.92, 0.59)	CON: -0.13<br>(-1.67, 1.32)<br>DRY: 0.65<br>(-1.42, 2.74)	CON: 0.25<br>(-5.27, 5.86)<br>DRY: -2.87<br>(-6.30, -0.11)	CON: 10.36<br>(7.06, 14.60)<br>DRY: 15.99<br>(9.85, 23.91)	
**C:N ratio**	CON: -0.31<br>(-0.84, 0.16)<br>DRY: -0.01<br>(-0.13, 0.10)	CON: -0.09<br>(-0.76, 0.58)<br>DRY: -0.43<br>(-0.93, 0.00)	CON: -0.10<br>(-0.51, 0.29)<br>DRY: -0.29<br>(-0.62, -0.02)	CON: -0.01<br>(-0.44, 0.41)<br>DRY: -0.20<br>(-0.49, 0.00)	CON: -0.04<br>(-0.17, 0.07)<br>DRY: 0.05<br>(-0.03, 0.15)	CON: 0.00<br>(-0.29, 0.30)<br>DRY: -0.12<br>(-0.42, 0.09)	CON: 0.23<br>(-1.26, 1.77)<br>DRY: 0.35<br>(0.00, 0.81)	CON: -0.44<br>(-1.20, 0.22)<br>DRY: -0.41<br>(-1.11, 0.13)	CON: 0.31<br>(0.06, 0.64)<br>DRY: 0.10<br>(0.00, 0.26)
**relative fitness**	CON: -0.16<br>(-0.28, -0.05)<br>DRY: 0.01<br>(-0.07, 0.09)	CON: -0.35<br>(-0.54, -0.20)<br>DRY: -0.61<br>(-0.91, -0.37)	CON: -0.17<br>(-0.28, -0.07)<br>DRY: -0.34<br>(-0.53, -0.20)	CON: -0.05<br>(-0.17, 0.07)<br>DRY: -0.24<br>(-0.42, -0.09)	CON: 0.03<br>(-0.01, 0.06)<br>DRY: 0.08<br>(0.02, 0.14)	CON: -0.04<br>(-0.17, 0.06)<br>DRY: -0.18<br>(-0.41, 0.00)	CON: 0.79<br>(0.42, 1.25)<br>DRY: 0.47<br>(0.21, 0.80)	CON: -0.01<br>(-0.18, 0.16)<br>DRY: -0.54<br>(-0.97, -0.19)	CON: 0.01<br>(-0.04, 0.06)<br>DRY: 0.06<br>(0.00, 0.12)

Table S11: *Trifolium pratense – G-Matrix of prior Specification G10vp2:* Posterior mean MCMC estimates of G for both treatments (CON & DRY), with 95% Bayesian Credible intervals in brackets. Traits with significant treatment effect in genetic variances coloured in yellow; significant genetic covariances between trait pairs except with relative fitness coloured in green; significant genetic covariances with relative fitness coloured in blue.

G10vp2	biomass	leaf length	leaf width	SLA	hair density	plant height	plant width	flowering time	C:N ratio
**biomass**	CON: 5.43<br>(3.63, 7.81)<br>DRY: 1.08<br>(0.72, 1.58)								
**leaf length**	CON: 2.96<br>(1.16, 5.16)<br>DRY: 0.48<br>(-0.30, 1.30)	CON: 8.77<br>(5.92, 12.55)<br>DRY: 7.90<br>(5.43, 11.20)							
**leaf width**	CON: 1.83<br>(0.67, 3.27)<br>DRY: 0.18<br>(-0.32, 0.70)	CON: 3.42<br>(1.83, 5.52)<br>DRY: 3.42<br>(2.01, 5.28)	CON: 3.54<br>(2.37, 5.13)<br>DRY: 3.20<br>(2.18, 4.55)						
**SLA**	CON: 0.61<br>(-1.08, 2.41)<br>DRY: 0.16<br>(-0.46, 0.82)	CON: 0.71<br>(-1.41, 2.97)<br>DRY: 1.89<br>(0.26, 3.81)	CON: 0.77<br>(-0.59, 2.22)<br>DRY: 1.46<br>(0.41, 2.77)	CON: 6.95<br>(4.63, 10.07)<br>DRY: 4.69<br>(3.10, 6.87)					
**hair density**	CON: -0.17<br>(-0.58, 0.21)<br>DRY: -0.02<br>(-0.23, 0.18)	CON: -0.60<br>(-1.17, -0.13)<br>DRY: -0.84<br>(-1.47, -0.31)	CON: -0.46<br>(-0.84, -0.15)<br>DRY: -0.54<br>(-0.96, -0.19)	CON: -0.14<br>(-0.61, 0.30)<br>DRY: -0.41<br>(-0.91, 0.03)	CON: 0.37<br>(0.25, 0.54)<br>DRY: 0.53<br>(0.35, 0.75)				
**plant height**	CON: 1.27<br>(-0.78, 3.54)<br>DRY: 0.72<br>(-0.07, 1.68)	CON: 0.90<br>(-1.80, 3.70)<br>DRY: 2.23<br>(0.05, 4.79)	CON: 0.43<br>(-1.26, 2.20)<br>DRY: 1.29<br>(-0.10, 2.99)	CON: 1.57<br>(-0.69, 4.32)<br>DRY: 2.00<br>(0.35, 4.16)	CON: -0.12<br>(-0.70, 0.43)<br>DRY: -0.42<br>(-1.05, 0.13)	CON: 10.32<br>(6.82, 15.14)<br>DRY: 7.79<br>(5.16, 11.49)			
**plant width**	CON: -4.67<br>(-10.13, 0.15)<br>DRY: 0.37<br>(-0.57, 1.45)	CON: -9.23<br>(-16.70, -3.13)<br>DRY: -3.31<br>(-6.26, -0.81)	CON: -5.77<br>(-10.48, -1.92)<br>DRY: -2.23<br>(-4.10, -0.64)	CON: 0.70<br>(-4.92, 6.90)<br>DRY: -0.72<br>(-2.72, 1.28)	CON: 0.73<br>(-0.54, 2.11)<br>DRY: 0.53<br>(-0.10, 1.24)	CON: 6.43<br>(-0.60, 14.85)<br>DRY: 0.95<br>(-1.54, 3.86)	CON: 62.84<br>(42.02, 90.97)<br>DRY: 11.24<br>(7.44, 16.51)		
**flowering time**	CON: 0.28<br>(-1.79, 2.35)<br>DRY: 0.23<br>(-1.07, 1.53)	CON: -0.75<br>(-3.42, 1.78)<br>DRY: 3.16<br>(-0.05, 6.74)	CON: -1.18<br>(-2.95, 0.42)<br>DRY: 1.12<br>(-0.96, 3.33)	CON: -0.10<br>(-2.56, 2.36)<br>DRY: 1.58<br>(-1.06, 4.39)	CON: 0.77<br>(0.26, 1.38)<br>DRY: -0.07<br>(-0.94, 0.82)	CON: 0.28<br>(-2.67, 3.53)<br>DRY: 0.00<br>(-3.64, 3.57)	CON: 0.44<br>(-6.64, 7.79)<br>DRY: -3.49<br>(-8.06, 0.55)	CON: 11.50<br>(7.98, 16.04)<br>DRY: 20.37<br>(13.79, 28.87)	
**C:N ratio**	CON: -0.30<br>(-0.94, 0.30)<br>DRY: -0.02<br>(-0.23, 0.17)	CON: -0.04<br>(-0.84, 0.77)<br>DRY: -0.39<br>(-1.01, 0.17)	CON: -0.06<br>(-0.57, 0.44)<br>DRY: -0.28<br>(-0.67, 0.07)	CON: -0.05<br>(-0.69, 0.62)<br>DRY: -0.21<br>(-0.68, 0.22)	CON: -0.05<br>(-0.21, 0.11)<br>DRY: 0.05<br>(-0.09, 0.19)	CON: -0.02<br>(-0.81, 0.76)<br>DRY: -0.11<br>(-0.71, 0.43)	CON: 0.13<br>(-1.92, 2.18)<br>DRY: 0.28<br>(-0.36, 0.96)	CON: -0.45<br>(-1.34, 0.39)<br>DRY: -0.49<br>(-1.47, 0.39)	CON: 0.61<br>(0.37, 0.96)<br>DRY: 0.38<br>(0.23, 0.60)
**relative fitness**	CON: -0.11<br>(-0.25, 0.02)<br>DRY: 0.04<br>(-0.06, 0.14)	CON: -0.33<br>(-0.55, -0.16)<br>DRY: -0.60<br>(-0.93, -0.33)	CON: -0.16<br>(-0.29, -0.05)<br>DRY: -0.33<br>(-0.53, -0.16)	CON: -0.03<br>(-0.19, 0.14)<br>DRY: -0.22<br>(-0.45, -0.02)	CON: 0.02<br>(-0.01, 0.06)<br>DRY: 0.07<br>(0.00, 0.14)	CON: -0.04<br>(-0.25, 0.16)<br>DRY: -0.15<br>(-0.44, 0.13)	CON: 0.76<br>(0.29, 1.32)<br>DRY: 0.51<br>(0.18, 0.92)	CON: -0.03<br>(-0.22, 0.17)<br>DRY: -0.57<br>(-1.05, -0.16)	CON: 0.01<br>(-0.05, 0.07)<br>DRY: 0.05<br>(-0.02, 0.13)

Table S12: *Trifolium pratense – G-Matrix of prior Specification G10vp2.2:* Posterior mean MCMC estimates of G for both treatments (CON & DRY), with 95% Bayesian Credible intervals in brackets. Traits with significant treatment effect in genetic variances coloured in yellow; significant genetic covariances between trait pairs except with relative fitness coloured in green; significant genetic covariances with relative fitness in blue.

G10vp2.2	biomass	leaf length	leaf width	SLA	hair density	plant height	plant width	flowering time	C:N ratio
**biomass**	CON: 3.46<br>(1.74, 5.80)<br>DRY: 0.58<br>(0.21, 1.07)								
**leaf length**	CON: 3.37<br>(1.62, 5.64)<br>DRY: 0.54<br>(-0.22, 1.41)	CON: 6.46<br>(3.75, 10.14)<br>DRY: 6.84<br>(4.30, 10.16)							
**leaf width**	CON: 2.06<br>(0.91, 3.54)<br>DRY: 0.14<br>(-0.34, 0.68)	CON: 3.25<br>(1.68, 5.37)<br>DRY: 3.68<br>(2.17, 5.60)	CON: 2.41<br>(1.30, 3.87)<br>DRY: 2.66<br>(1.59, 4.06)						
**SLA**	CON: 0.59<br>(-0.92, 2.22)<br>DRY: 0.06<br>(-0.46, 0.65)	CON: 0.91<br>(-0.99, 2.93)<br>DRY: 2.43<br>(0.92, 4.35)	CON: 0.68<br>(-0.51, 2.05)<br>DRY: 1.64<br>(0.65, 2.94)	CON: 2.23<br>(0.62, 4.80)<br>DRY: 1.73<br>(0.64, 3.34)					
**hair density**	CON: -0.16<br>(-0.56, 0.20)<br>DRY: -0.01<br>(-0.22, 0.20)	CON: -0.62<br>(-1.18, -0.16)<br>DRY: -1.00<br>(-1.68, -0.46)	CON: -0.43<br>(-0.80, -0.13)<br>DRY: -0.58<br>(-1.02, -0.23)	CON: 0.04<br>(-0.39, 0.42)<br>DRY: -0.38<br>(-0.86, 0.00)	CON: 0.24<br>(0.12, 0.40)<br>DRY: 0.40<br>(0.22, 0.62)				
**plant height**	CON: 0.75<br>(-0.97, 2.82)<br>DRY: 0.36<br>(-0.27, 1.22)	CON: 1.16<br>(-1.10, 3.56)<br>DRY: 2.70<br>(0.72, 5.15)	CON: 0.36<br>(-1.04, 1.89)<br>DRY: 1.37<br>(0.10, 2.92)	CON: 0.37<br>(-1.11, 2.20)<br>DRY: 1.07<br>(-0.05, 2.72)	CON: -0.13<br>(-0.62, 0.33)<br>DRY: -0.53<br>(-1.13, -0.03)	CON: 2.12<br>(0.52, 4.91)<br>DRY: 2.37<br>(0.74, 4.92)			
**plant width**	CON: -5.85<br>(-11.19, -1.24)<br>DRY: 0.16<br>(-0.65, 1.09)	CON: -11.39<br>(-18.70, -5.55)<br>DRY: -4.02<br>(-6.88, -1.68)	CON: -7.30<br>(-11.88, -3.50)<br>DRY: -2.92<br>(-4.73, -1.47)	CON: -1.90<br>(-6.67, 3.28)<br>DRY: -1.86<br>(-3.37, -0.53)	CON: 0.99<br>(-0.24, 2.29)<br>DRY: 0.69<br>(0.10, 1.37)	CON: 0.90<br>(-4.48, 8.06)<br>DRY: -0.80<br>(-2.49, 1.19)	CON: 39.73<br>(20.33, 65.71)<br>DRY: 4.99<br>(2.09, 9.20)		
**flowering time**	CON: 0.31<br>(-1.75, 2.42)<br>DRY: 0.39<br>(-0.92, 1.73)	CON: -1.09<br>(-3.74, 1.48)<br>DRY: 3.85<br>(0.55, 7.59)	CON: -1.44<br>(-3.24, 0.11)<br>DRY: 1.45<br>(-0.69, 3.70)	CON: -0.19<br>(-2.46, 2.11)<br>DRY: 2.38<br>(-0.16, 5.13)	CON: 1.00<br>(0.47, 1.64)<br>DRY: -0.16<br>(-1.05, 0.73)	CON: -0.60<br>(-3.34, 2.22)<br>DRY: 0.06<br>(-3.34, 3.38)	CON: -0.52<br>(-7.44, 6.52)<br>DRY: -3.83<br>(-8.43, -0.01)	CON: 10.65<br>(7.12, 15.43)<br>DRY: 17.06<br>(10.26, 25.90)	
**C:N ratio**	CON: -0.53<br>(-1.21, 0.09)<br>DRY: -0.02<br>(-0.20, 0.16)	CON: -0.13<br>(-0.94, 0.67)<br>DRY: -0.54<br>(-1.15, 0.00)	CON: -0.16<br>(-0.67, 0.31)<br>DRY: -0.42<br>(-0.81, -0.10)	CON: 0.06<br>(-0.53, 0.66)<br>DRY: -0.32<br>(-0.71, -0.02)	CON: -0.06<br>(-0.22, 0.09)<br>DRY: 0.05<br>(-0.08, 0.19)	CON: 0.02<br>(-0.61, 0.62)<br>DRY: -0.11<br>(-0.54, 0.30)	CON: 0.25<br>(-1.65, 2.23)<br>DRY: 0.53<br>(0.06, 1.09)	CON: -0.68<br>(-1.57, 0.14)<br>DRY: -0.68<br>(-1.65, 0.18)	CON: 0.32<br>(0.10, 0.65)<br>DRY: 0.15<br>(0.04, 0.33)
**Relative fitness**	CON: -0.20<br>(-0.35, -0.07)<br>DRY: 0.02<br>(-0.08, 0.12)	CON: -0.43<br>(-0.65, -0.26)<br>DRY: -0.72<br>(-1.07, -0.45)	CON: -0.20<br>(-0.33, -0.09)<br>DRY: -0.41<br>(-0.63, -0.24)	CON: -0.07<br>(-0.22, 0.08)<br>DRY: -0.31<br>(-0.53, -0.12)	CON: 0.03<br>(0.00, 0.07)<br>DRY: 0.09<br>(0.03, 0.17)	CON: -0.07<br>(-0.25, 0.11)<br>DRY: -0.24<br>(-0.51, 0.00)	CON: 0.92<br>(0.47, 1.48)<br>DRY: 0.54<br>(0.24, 0.94)	CON: -0.02<br>(-0.22, 0.17)<br>DRY: -0.67<br>(-1.17, -0.25)	CON: 0.01<br>(-0.05, 0.07)<br>DRY: 0.07<br>(0.01, 0.15)

Table S13: *Trifolium pratense – G-Matrix of prior Specification G10e3:* Posterior mean MCMC estimates of G for both treatments (CON & DRY), with 95% Bayesian Credible intervals in brackets. Traits with significant treatment effect in genetic variances coloured in yellow; significant genetic covariances between trait pairs except with relative fitness coloured in green; significant genetic covariances with relative fitness coloured in blue.

G10e3	biomass	leaf length	leaf width	SLA	hair density	plant height	plant width	flowering time	C:N ratio
**biomass**	CON: 3.06<br>(1.49, 5.08)<br>DRY: 0.48<br>(0.16, 0.87)								
**leaf length**	CON: 2.66<br>(1.18, 4.53)<br>DRY: 0.29<br>(-0.34, 1.03)	CON: 5.68<br>(3.22, 8.87)<br>DRY: 5.71<br>(3.51, 8.54)							
**leaf width**	CON: 1.57<br>(0.63, 2.76)<br>DRY: 0.07<br>(-0.34, 0.51)	CON: 2.67<br>(1.33, 4.42)<br>DRY: 2.99<br>(1.71, 4.67)	CON: 2.05<br>(1.04, 3.34)<br>DRY: 2.21<br>(1.31, 3.44)						
**SLA**	CON: 0.40<br>(-0.75, 1.65)<br>DRY: 0.03<br>(-0.33, 0.41)	CON: 0.61<br>(-0.95, 2.30)<br>DRY: 1.85<br>(0.57, 3.45)	CON: 0.48<br>(-0.43, 1.59)<br>DRY: 1.23<br>(0.40, 2.31)	CON: 2.22<br>(0.07, 5.04)<br>DRY: 1.31<br>(0.30, 2.83)					
**hair density**	CON: -0.13<br>(-0.46, 0.17)<br>DRY: 0.01<br>(-0.15, 0.17)	CON: -0.49<br>(-0.97, -0.10)<br>DRY: -0.79<br>(-1.36, -0.33)	CON: -0.34<br>(-0.66, -0.09)<br>DRY: -0.46<br>(-0.83, -0.16)	CON: -0.01<br>(-0.33, 0.29)<br>DRY: -0.28<br>(-0.64, 0.00)	CON: 0.24<br>(0.13, 0.38)<br>DRY: 0.37<br>(0.21, 0.58)				
**plant height**	CON: 0.31<br>(-0.58, 1.61)<br>DRY: 0.12<br>(-0.22, 0.63)	CON: 0.54<br>(-0.80, 2.35)<br>DRY: 1.71<br>(0.01, 3.78)	CON: 0.25<br>(-0.56, 1.29)<br>DRY: 0.96<br>(-0.03, 2.24)	CON: 0.12<br>(-0.53, 1.04)<br>DRY: 0.67<br>(-0.07, 1.93)	CON: -0.07<br>(-0.38, 0.16)<br>DRY: -0.31<br>(-0.76, 0.01)	CON: 0.73<br>(0.00, 3.14)<br>DRY: 1.29<br>(0.03, 3.45)			
**plant width**	CON: -5.46<br>(-9.67, -1.96)<br>DRY: 0.00<br>(-0.56, 0.65)	CON: -9.94<br>(-15.87, -5.04)<br>DRY: -3.65<br>(-6.04, -1.70)	CON: -5.91<br>(-9.67, -2.88)<br>DRY: -2.39<br>(-3.92, -1.17)	CON: -1.50<br>(-5.39, 2.26)<br>DRY: -1.48<br>(-2.69, -0.48)	CON: 0.84<br>(-0.12, 1.93)<br>DRY: 0.59<br>(0.16, 1.13)	CON: -0.46<br>(-3.66, 3.31)<br>DRY: -1.00<br>(-2.20, 0.24)	CON: 35.07<br>(18.80, 55.51)<br>DRY: 4.01<br>(1.34, 7.75)		
**flowering time**	CON: 0.29<br>(-1.42, 2.07)<br>DRY: 0.43<br>(-0.57, 1.48)	CON: -0.77<br>(-3.01, 1.43)<br>DRY: 3.58<br>(0.82, 6.69)	CON: -1.01<br>(-2.49, 0.36)<br>DRY: 1.44<br>(-0.33, 3.35)	CON: 0.09<br>(-1.71, 1.82)<br>DRY: 1.81<br>(0.16, 3.86)	CON: 0.76<br>(0.30, 1.31)<br>DRY: -0.19<br>(-0.93, 0.56)	CON: -0.16<br>(-1.74, 1.23)<br>DRY: 0.64<br>(-1.44, 2.81)	CON: 0.26<br>(-5.31, 5.79)<br>DRY: -2.82<br>(-6.19, -0.09)	CON: 10.31<br>(6.94, 14.67)<br>DRY: 15.92<br>(9.79, 23.95)	
**C:N ratio**	CON: -0.31<br>(-0.84, 0.15)<br>DRY: -0.01<br>(-0.13, 0.10)	CON: -0.10<br>(-0.77, 0.56)<br>DRY: -0.42<br>(-0.94, 0.00)	CON: -0.10<br>(-0.51, 0.29)<br>DRY: -0.29<br>(-0.61, -0.01)	CON: -0.01<br>(-0.42, 0.41)<br>DRY: -0.20<br>(-0.50, 0.00)	CON: -0.04<br>(-0.17, 0.07)<br>DRY: 0.05<br>(-0.03, 0.15)	CON: 0.00<br>(-0.29, 0.30)<br>DRY: -0.12<br>(-0.43, 0.09)	CON: 0.24<br>(-1.31, 1.80)<br>DRY: 0.35<br>(0.00, 0.80)	CON: -0.44<br>(-1.17, 0.21)<br>DRY: -0.40<br>(-1.14, 0.11)	CON: 0.30<br>(0.05, 0.62)<br>DRY: 0.10<br>(0.00, 0.27)
**Relative fitness**	CON: -0.16<br>(-0.28, -0.05)<br>DRY: 0.01<br>(-0.07, 0.09)	CON: -0.35<br>(-0.53, -0.20)<br>DRY: -0.61<br>(-0.90, -0.38)	CON: -0.17<br>(-0.27, -0.08)<br>DRY: -0.34<br>(-0.52, -0.20)	CON: -0.05<br>(-0.17, 0.07)<br>DRY: -0.24<br>(-0.43, -0.08)	CON: 0.03<br>(0.00, 0.06)<br>DRY: 0.08<br>(0.02, 0.14)	CON: -0.04<br>(-0.17, 0.06)<br>DRY: -0.19<br>(-0.41, 0.01)	CON: 0.79<br>(0.42, 1.24)<br>DRY: 0.46<br>(0.20, 0.80)	CON: -0.01<br>(-0.18, 0.16)<br>DRY: -0.53<br>(-0.95, -0.18)	CON: 0.01<br>(-0.04, 0.06)<br>DRY: 0.05<br>(0.00, 0.12)

Table S14: G-Matrix comparison statistics: Descriptors of multivariate genetic variation – number of dimensions n_D, maximum evolvability e_{max}, and total genetic variance *tgv* for each treatment. Significant treatment effects (CI does not overlap other treatment mean) are coloured in blue.

**Matrix comparison statistics**	***Bromus erectus***		***Trifolium pratense***	
	Mode	95% CI	Mode	95% CI
Number of dimensions n_D (Control)	1.74	(1.45, 2.05)	1.55	(1.31, 1.89)
Number of dimensions n_D (Drought)	1.68	(1.40, 2.00)	1.20	(1.11, 1.34)
Maximum evolvability e_{max} (Control)	0.45	(0.38, 0.55)	0.23	(0.19, 0.28)
Maximum evolvability e_{max} (Drought)	0.42	(0.35, 0.50)	0.33	(0.26, 0.40)
Total genetic variance *tgv* (Drought)	0.36	(0.26, 0.48)	0.08	(0.06, 0.11)
Total genetic variance *tgv* (Control)	0.29	(0.21, 0.39)	0.13	(0.09, 0.18)

Table S15: *Bromus erectus* – Quantitative trait heritabilities H^2 for all prior specifications (choice in bold): Posterior mean MCMC estimates for both treatments (CON & DRY), with 95% Bayesian Credible intervals in brackets. Trait estimates with significant treatment effect coloured in blue.

***Bromus erectus* H^2**	G11vp2	G11vp2.2	**G11e2**	G11e3
**vegetative biomass**	CON: 0.26<br>(0.19, 0.34)<br>DRY: 0.34<br>(0.25, 0.43)	CON: 0.16<br>(0.08, 0.26)<br>DRY: 0.28<br>(0.18, 0.39)	**CON: 0.17<br>(0.08, 0.26)<br>DRY: 0.29<br>(0.19, 0.39)**	CON: 0.16<br>(0.08, 0.26)<br>DRY: 0.29<br>(0.19, 0.39)
**leaf length**	CON: 0.24<br>(0.18, 0.32)<br>DRY: 0.24<br>(0.17, 0.31)	CON: 0.13<br>(0.06, 0.22)<br>DRY: 0.08<br>(0.03, 0.16)	**CON: 0.12<br>(0.04, 0.21)<br>DRY: 0.07<br>(0.00, 0.16)**	CON: 0.12<br>(0.04, 0.20)<br>DRY: 0.07<br>(0.00, 0.16)
**leaf width**	CON: 0.26<br>(0.18, 0.34)<br>DRY: 0.21<br>(0.15, 0.29)	CON: 0.16<br>(0.08, 0.25)<br>DRY: 0.08<br>(0.03, 0.15)	**CON: 0.16<br>(0.08, 0.25)<br>DRY: 0.07<br>(0.01, 0.16)**	CON: 0.16<br>(0.08, 0.25)<br>DRY: 0.07<br>(0.01, 0.16)
**SLA**	CON: 0.29<br>(0.21, 0.38)<br>DRY: 0.23<br>(0.17, 0.31)	CON: 0.22<br>(0.12, 0.32)<br>DRY: 0.11<br>(0.04, 0.21)	**CON: 0.21<br>(0.12, 0.31)<br>DRY: 0.12<br>(0.04, 0.21)**	CON: 0.21<br>(0.12, 0.31)<br>DRY: 0.12<br>(0.04, 0.21)
**C:N ratio**	CON: 0.35<br>(0.24, 0.47)<br>DRY: 0.29<br>(0.20, 0.41)	CON: 0.27<br>(0.13, 0.42)<br>DRY: 0.18<br>(0.08, 0.31)	**CON: 0.26<br>(0.13, 0.40)<br>DRY: 0.16<br>(0.05, 0.29)**	CON: 0.26<br>(0.13, 0.40)<br>DRY: 0.16<br>(0.05, 0.29)
**hair density**	CON: 0.36<br>(0.28, 0.45)<br>DRY: 0.37<br>(0.29, 0.47)	CON: 0.32<br>(0.22, 0.42)<br>DRY: 0.34<br>(0.23, 0.44)	**CON: 0.32<br>(0.22, 0.42)<br>DRY: 0.34<br>(0.25, 0.44)**	CON: 0.32<br>(0.22, 0.42)<br>DRY: 0.34<br>(0.25, 0.44)
**plant height**	CON: 0.23<br>(0.16, 0.31)<br>DRY: 0.25<br>(0.18, 0.34)	CON: 0.11<br>(0.04, 0.20)<br>DRY: 0.14<br>(0.05, 0.24)	**CON: 0.08<br>(0.01, 0.15)<br>DRY: 0.10<br>(0.02, 0.20)**	CON: 0.08<br>(0.01, 0.15)<br>DRY: 0.10<br>(0.02, 0.19)
**inflorescence length**	CON: 0.20<br>(0.14, 0.27)<br>DRY: 0.23<br>(0.16, 0.31)	CON: 0.06<br>(0.02, 0.14)<br>DRY: 0.11<br>(0.03, 0.20)	**CON: 0.02<br>(0.00, 0.09)<br>DRY: 0.08<br>(0.00, 0.18)**	CON: 0.02<br>(0.00, 0.09)<br>DRY: 0.08<br>(0.00, 0.18)
**stem weight**	CON: 0.23<br>(0.16, 0.31)<br>DRY: 0.27<br>(0.20, 0.36)	CON: 0.11<br>(0.04, 0.21)<br>DRY: 0.18<br>(0.09, 0.29)	**CON: 0.07<br>(0.01, 0.15)<br>DRY: 0.13<br>(0.04, 0.23)**	CON: 0.07<br>(0.01, 0.15)<br>DRY: 0.13<br>(0.04, 0.24)
**start of flowering**	CON: 0.27<br>(0.19, 0.35)<br>DRY: 0.25<br>(0.18, 0.34)	CON: 0.17<br>(0.08, 0.26)<br>DRY: 0.13<br>(0.05, 0.22)	**CON: 0.17<br>(0.09, 0.27)<br>DRY: 0.14<br>(0.05, 0.24)**	CON: 0.17<br>(0.08, 0.27)<br>DRY: 0.14<br>(0.05, 0.25)
**relative fitness**	CON: 0.29<br>(0.21, 0.38)<br>DRY: 0.31<br>(0.23, 0.40)	CON: 0.22<br>(0.13, 0.32)<br>DRY: 0.26<br>(0.16, 0.36)	**CON: 0.22<br>(0.13, 0.32)<br>DRY: 0.25<br>(0.16, 0.35)**	CON: 0.22<br>(0.13, 0.32)<br>DRY: 0.25<br>(0.16, 0.35)

Table S16: *Trifolium pratense* – Quantitative trait heritabilities H² for all prior specifications (choice in bold): Posterior mean MCMC estimates for both treatments (CON & DRY), with 95% Bayesian Credible intervals in brackets. Trait estimates with significant treatment effect coloured in blue.

***Trifolium pratense* H²**	G10vp2	G10vp2.2	**G10e2**	G10e3
**biomass**	CON: 0.26<br>(0.19, 0.35)<br>DRY: 0.22<br>(0.15, 0.29)	CON: 0.18<br>(0.09, 0.27)<br>DRY: 0.12<br>(0.05, 0.21)	**CON: 0.16**<br>**(0.08, 0.25)**<br>**DRY: 0.11**<br>**(0.04, 0.18)**	CON: 0.16<br>(0.08, 0.25)<br>DRY: 0.10<br>(0.04, 0.18)
**leaf length**	CON: 0.29<br>(0.21, 0.38)<br>DRY: 0.34<br>(0.25, 0.43)	CON: 0.22<br>(0.14, 0.32)<br>DRY: 0.30<br>(0.21, 0.40)	**CON: 0.21**<br>**(0.12, 0.30)**<br>**DRY: 0.26**<br>**(0.18, 0.36)**	CON: 0.20<br>(0.12, 0.29)<br>DRY: 0.26<br>(0.17, 0.36)
**leaf width**	CON: 0.27<br>(0.19, 0.36)<br>DRY: 0.31<br>(0.22, 0.39)	CON: 0.19<br>(0.11, 0.29)<br>DRY: 0.26<br>(0.17, 0.36)	**CON: 0.17**<br>**(0.09, 0.26)**<br>**DRY: 0.23**<br>**(0.14, 0.32)**	CON: 0.17<br>(0.09, 0.26)<br>DRY: 0.23<br>(0.14, 0.33)
**SLA**	CON: 0.20<br>(0.14, 0.27)<br>DRY: 0.18<br>(0.13, 0.25)	CON: 0.07<br>(0.02, 0.14)<br>DRY: 0.07<br>(0.03, 0.13)	**CON: 0.07**<br>**(0.00, 0.15)**<br>**DRY: 0.06**<br>**(0.01, 0.12)**	CON: 0.07<br>(0.00, 0.15)<br>DRY: 0.06<br>(0.01, 0.12)
**C:N ratio**	CON: 0.28<br>(0.18, 0.39)<br>DRY: 0.23<br>(0.15, 0.34)	CON: 0.15<br>(0.05, 0.29)<br>DRY: 0.11<br>(0.03, 0.21)	**CON: 0.15**<br>**(0.03, 0.29)**<br>**DRY: 0.07**<br>**(0.00, 0.18)**	CON: 0.15<br>(0.03, 0.28)<br>DRY: 0.07<br>(0.00, 0.18)
**hair density**	CON: 0.27<br>(0.20, 0.36)<br>DRY: 0.28<br>(0.20, 0.37)	CON: 0.18<br>(0.10, 0.28)<br>DRY: 0.22<br>(0.13, 0.32)	**CON: 0.19**<br>**(0.11, 0.28)**<br>**DRY: 0.21**<br>**(0.13, 0.31)**	CON: 0.19<br>(0.11, 0.28)<br>DRY: 0.21<br>(0.13, 0.31)
**plant height**	CON: 0.16<br>(0.11, 0.23)<br>DRY: 0.16<br>(0.11, 0.22)	CON: 0.04<br>(0.01, 0.08)<br>DRY: 0.05<br>(0.02, 0.10)	**CON: 0.01**<br>**(0.00, 0.05)**<br>**DRY: 0.03**<br>**(0.00, 0.07)**	CON: 0.01<br>(0.00, 0.05)<br>DRY: 0.03<br>(0.00, 0.07)
**plant width**	CON: 0.25<br>(0.18, 0.33)<br>DRY: 0.19<br>(0.13, 0.25)	CON: 0.16<br>(0.09, 0.25)<br>DRY: 0.09<br>(0.04, 0.16)	**CON: 0.15**<br>**(0.08, 0.22)**<br>**DRY: 0.07**<br>**(0.03, 0.14)**	CON: 0.15<br>(0.08, 0.22)<br>DRY: 0.07<br>(0.03, 0.14)
**start of flowering**	CON: 0.41<br>(0.32, 0.51)<br>DRY: 0.33<br>(0.24, 0.42)	CON: 0.39<br>(0.29, 0.49)<br>DRY: 0.28<br>(0.18, 0.39)	**CON: 0.39**<br>**(0.29, 0.48)**<br>**DRY: 0.27**<br>**(0.18, 0.37)**	CON: 0.38<br>(0.29, 0.48)<br>DRY: 0.27<br>(0.18, 0.37)
**relative fitness**	CON: 0.32<br>(0.24, 0.41)<br>DRY: 0.31<br>(0.23, 0.40)	CON: 0.26<br>(0.17, 0.36)<br>DRY: 0.26<br>(0.17, 0.36)	**CON: 0.25**<br>**(0.17, 0.35)**<br>**DRY: 0.23**<br>**(0.15, 0.33)**	CON: 0.25<br>(0.17, 0.35)<br>DRY: 0.23<br>(0.15, 0.32)

Table S17: *Bromus erectus* – Evolutionary response Δz for all prior specifications (choice in bold): Posterior mean MCMC estimates for both treatments (CON & DRY), with 95% Bayesian Credible intervals in brackets. Significant evolutionary responses coloured in blue.

***Bromus erectus* Δz**	G11vp2	G11vp2.2	**G11e2**	G11e3
**vegetative biomass**	CON: -0.04 (-0.20, 0.11) DRY: -0.02 (-0.12, 0.08)	CON: -0.06 (-0.22, 0.10) DRY: -0.03 (-0.13, 0.06)	**CON: -0.04 (-0.17, 0.08) DRY: -0.02 (-0.10, 0.07)**	CON: -0.04 (-0.17, 0.09) DRY: -0.02 (-0.10, 0.07)
**leaf length**	CON: -0.01 (-0.29, 0.28) DRY: 0.03 (-0.16, 0.23)	CON: -0.03 (-0.30, 0.23) DRY: 0.05 (-0.14, 0.25)	**CON: 0.00 (-0.22, 0.22) DRY: 0.03 (-0.11, 0.17)**	CON: 0.00 (-0.21, 0.22) DRY: 0.03 (-0.11, 0.17)
**leaf width**	CON: -0.02 (-0.06, 0.03) DRY: -0.01 (-0.04, 0.02)	CON: -0.03 (-0.07, 0.02) DRY: -0.02 (-0.04, 0.01)	**CON: -0.02 (-0.06, 0.02) DRY: -0.01 (-0.03, 0.01)**	CON: -0.02 (-0.06, 0.02) DRY: -0.01 (-0.04, 0.01)
**SLA**	CON: -0.06 (-0.25, 0.12) DRY: -0.03 (-0.16, 0.10)	CON: -0.07 (-0.26, 0.11) DRY: -0.02 (-0.15, 0.10)	**CON: -0.06 (-0.23, 0.09) DRY: -0.02 (-0.13, 0.08)**	CON: -0.06 (-0.22, 0.09) DRY: -0.02 (-0.13, 0.08)
**C:N ratio**	CON: -0.25 (-0.71, 0.18) DRY: -0.25 (-0.58, 0.03)	CON: -0.43 (-0.94, 0.03) DRY: -0.37 (-0.72, -0.08)	**CON: -0.27 (-0.68, 0.10) DRY: -0.23 (-0.51, -0.01)**	CON: -0.27 (-0.69, 0.09) DRY: -0.23 (-0.51, -0.01)
**hair density**	CON: 0.40 (-0.57, 1.40) DRY: 0.56 (-0.38, 1.57)	CON: 0.59 (-0.40, 1.62) DRY: 0.76 (-0.20, 1.81)	**CON: 0.46 (-0.38, 1.34) DRY: 0.64 (-0.21, 1.56)**	CON: 0.46 (-0.37, 1.34) DRY: 0.63 (-0.20, 1.52)
**plant height**	CON: -0.14 (-0.87, 0.61) DRY: -0.49 (-1.31, 0.24)	CON: -0.39 (-1.11, 0.34) DRY: -0.70 (-1.54, 0.05)	**CON: -0.15 (-0.68, 0.34) DRY: -0.47 (-1.14, 0.09)**	CON: -0.15 (-0.69, 0.35) DRY: -0.47 (-1.12, 0.07)
**inflorescence length**	CON: 0.00 (-0.10, 0.11) DRY: 0.02 (-0.09, 0.13)	CON: -0.01 (-0.11, 0.08) DRY: 0.01 (-0.09, 0.12)	**CON: 0.00 (-0.06, 0.05) DRY: 0.01 (-0.07, 0.09)**	CON: 0.00 (-0.05, 0.06) DRY: 0.01 (-0.07, 0.09)
**stem weight**	CON: -0.01 (-0.02, 0.01) DRY: -0.01 (-0.02, 0.00)	CON: -0.01 (-0.02, 0.00) DRY: -0.01 (-0.02, 0.00)	**CON: 0.00 (-0.01, 0.00) DRY: -0.01 (-0.01, 0.00)**	CON: 0.00 (-0.01, 0.00) DRY: -0.01 (-0.01, 0.00)
**start of flowering**	CON: 0.33 (0.05, 0.65) DRY: 0.07 (-0.21, 0.35)	CON: 0.49 (0.22, 0.81) DRY: 0.08 (-0.19, 0.37)	**CON: 0.34 (0.12, 0.61) DRY: 0.07 (-0.15, 0.31)**	CON: 0.34 (0.11, 0.61) DRY: 0.08 (-0.15, 0.32)

Table S18: *Trifolium pratense* – Evolutionary response Δz for all prior specifications (choice in bold): Posterior mean MCMC estimates for both treatments (CON & DRY), with 95% Bayesian Credible intervals in brackets. Significant evolutionary responses coloured in blue.

***Trifolium pratense* Δz**	G10vp2	G10vp2.2	**G10e2**	G10e3
**biomass**	CON: -0.11 (-0.25, 0.02)	CON: -0.20 (-0.35, -0.07)	**CON: -0.16 (-0.28, -0.05)**	CON: -0.16 (-0.28, -0.05)
	DRY: 0.04 (-0.06, 0.14)	DRY: 0.02 (-0.08, 0.12)	**DRY: 0.01 (-0.07, 0.09)**	DRY: 0.01 (-0.07, 0.09)
**leaf length**	CON: -0.33 (-0.55, -0.16)	CON: -0.43 (-0.65, -0.26)	**CON: -0.35 (-0.54, -0.20)**	CON: -0.35 (-0.53, -0.20)
	DRY: -0.60 (-0.93, -0.33)	DRY: -0.72 (-1.07, -0.45)	**DRY: -0.61 (-0.91, -0.37)**	DRY: -0.61 (-0.90, -0.38)
**leaf width**	CON: -0.16 (-0.29, -0.05)	CON: -0.20 (-0.33, -0.09)	**CON: -0.17 (-0.28, -0.07)**	CON: -0.17 (-0.27, -0.08)
	DRY: -0.33 (-0.53, -0.16)	DRY: -0.41 (-0.63, -0.24)	**DRY: -0.34 (-0.53, -0.20)**	DRY: -0.34 (-0.52, -0.20)
**SLA**	CON: -0.03 (-0.19, 0.14)	CON: -0.07 (-0.22, 0.08)	**CON: -0.05 (-0.17, 0.07)**	CON: -0.05 (-0.17, 0.07)
	DRY: -0.22 (-0.45, -0.02)	DRY: -0.31 (-0.53, -0.12)	**DRY: -0.24 (-0.42, -0.09)**	DRY: -0.24 (-0.43, -0.08)
**C:N ratio**	CON: 0.01 (-0.05, 0.07)	CON: 0.01 (-0.05, 0.07)	**CON: 0.01 (-0.04, 0.06)**	CON: 0.01 (-0.04, 0.06)
	DRY: 0.05 (-0.02, 0.13)	DRY: 0.07 (0.01, 0.15)	**DRY: 0.06 (0.00, 0.12)**	DRY: 0.06 (0.00, 0.12)
**hair density**	CON: 0.02 (-0.01, 0.06)	CON: 0.03 (0.00, 0.07)	**CON: 0.03 (-0.01, 0.06)**	CON: 0.03 (0.00, 0.06)
	DRY: 0.07 (0.00, 0.14)	DRY: 0.09 (0.03, 0.17)	**DRY: 0.08 (0.02, 0.14)**	DRY: 0.08 (0.02, 0.14)
**plant height**	CON: -0.04 (-0.25, 0.16)	CON: -0.07 (-0.25, 0.11)	**CON: -0.04 (-0.17, 0.06)**	CON: -0.04 (-0.17, 0.06)
	DRY: -0.15 (-0.44, 0.13)	DRY: -0.24 (-0.51, 0.00)	**DRY: -0.18 (-0.41, 0.00)**	DRY: -0.19 (-0.41, 0.01)
**plant width**	CON: 0.76 (0.29, 1.32)	CON: 0.92 (0.47, 1.48)	**CON: 0.79 (0.42, 1.25)**	CON: 0.79 (0.42, 1.24)
	DRY: 0.51 (0.18, 0.92)	DRY: 0.54 (0.24, 0.94)	**DRY: 0.47 (0.21, 0.80)**	DRY: 0.46 (0.20, 0.80)
**start of flowering**	CON: -0.03 (-0.22, 0.17)	CON: -0.02 (-0.22, 0.17)	**CON: -0.01 (-0.18, 0.16)**	CON: -0.01 (-0.18, 0.16)
	DRY: -0.57 (-1.06, -0.16)	DRY: -0.67 (-1.17, -0.25)	**DRY: -0.54 (-0.97, -0.19)**	DRY: -0.53 (-0.95, -0.18)

Table S19: *Bromus erectus* – Selection gradients β for all prior specifications (choice in bold): Posterior mean MCMC estimates for both treatments (CON & DRY), with 95% Bayesian Credible intervals in brackets. None of the functional traits revealed significant selection gradients or treatment effects.

***Bromus erectus* β**	G11vp2	G11vp2.2	**G11e2**	G11e3
**vegetative biomass**	CON: -0.01<br>(-0.12, 0.09)<br>DRY: -0.04<br>(-0.17, 0.09)	CON: 0.02<br>(-0.44, 0.50)<br>DRY: -0.17<br>(-0.74, 0.42)	**CON: -0.01**<br>**(-0.20, 0.18)**<br>**DRY: -0.08**<br>**(-0.26, 0.10)**	CON: -0.01<br>(-0.20, 0.18)<br>DRY: -0.08<br>(-0.26, 0.10)
**leaf length**	CON: -0.01<br>(-0.06, 0.05)<br>DRY: 0.02<br>(-0.04, 0.08)	CON: -0.03<br>(-0.30, 0.24)<br>DRY: 0.09<br>(-0.32, 0.49)	**CON: -0.02**<br>**(-0.18, 0.12)**<br>**DRY: 0.12**<br>**(-0.28, 0.51)**	CON: -0.02<br>(-0.17, 0.12)<br>DRY: -0.06<br>(-0.27, 0.47)
**leaf width**	CON: -0.03<br>(-0.40, 0.34)<br>DRY: -0.03<br>(-0.47, 0.40)	CON: -0.07<br>(-1.76, 1.70)<br>DRY: 0.12<br>(-2.96, 3.28)	**CON: -0.03**<br>**(-0.84, 0.75)**<br>**DRY: -0.16**<br>**(-2.32, 1.68)**	CON: -0.03<br>(-0.87, 0.76)<br>DRY: -0.18<br>(-2.27, 1.66)
**SLA**	CON: -0.03<br>(-0.11, 0.06)<br>DRY: -0.04<br>(-0.13, 0.07)	CON: -0.04<br>(-0.40, 0.32)<br>DRY: -0.10<br>(-0.74, 0.52)	**CON: -0.03**<br>**(-0.17, 0.11)**<br>**DRY: -0.06**<br>**(-0.31, 0.17)**	CON: -0.03<br>(-0.17, 0.11)<br>DRY: -0.06<br>(-0.30, 0.18)
**C:N ratio**	CON: -0.02<br>(-0.07, 0.03)<br>DRY: -0.04<br>(-0.10, 0.02)	CON: -0.05<br>(-0.25, 0.15)<br>DRY: -0.15<br>(-0.48, 0.23)	**CON: 0.00**<br>**(-0.11, 0.05)**<br>**DRY: -0.08**<br>**(-0.25, 0.08)**	CON: -0.03<br>(-0.11, 0.05)<br>DRY: -0.07<br>(-0.26, 0.09)
**hair density**	CON: 0.00<br>(-0.01, 0.02)<br>DRY: 0.01<br>(-0.01, 0.02)	CON: 0.01<br>(-0.04, 0.05)<br>DRY: 0.00<br>(-0.06, 0.06)	**CON: 0.00**<br>**(-0.01, 0.02)**<br>**DRY: 0.00**<br>**(-0.01, 0.02)**	CON: 0.01<br>(-0.01, 0.02)<br>DRY: 0.00<br>(-0.01, 0.02)
**plant height**	CON: 0.00<br>(-0.03, 0.02)<br>DRY: -0.01<br>(-0.03, 0.01)	CON: -0.03<br>(-0.14, 0.07)<br>DRY: -0.03<br>(-0.15, 0.09)	**CON: -0.01**<br>**(-0.11, 0.06)**<br>**DRY: -0.02**<br>**(-0.10, 0.04)**	CON: -0.02<br>(-0.11, 0.05)<br>DRY: -0.02<br>(-0.10, 0.04)
**inflorescence length**	CON: 0.03<br>(-0.14, 0.19)<br>DRY: 0.06<br>(-0.07, 0.20)	CON: 0.00<br>(-0.86, 0.90)<br>DRY: 0.28<br>(-0.53, 1.10)	**CON: -0.43**<br>**(-5.34, 5.51)**<br>**DRY: 0.16**<br>**(-0.52, 0.89)**	CON: -0.60<br>(-5.26, 5.78)<br>DRY: 0.22<br>(-0.52, 0.93)
**stem weight**	CON: -0.65<br>(-2.19, 0.91)<br>DRY: -0.61<br>(-2.02, 0.80)	CON: -1.15<br>(-8.63, 6.50)<br>DRY: -0.55<br>(-9.44, 8.71)	**CON: -1.74**<br>**(-8.67, 4.26)**<br>**DRY: -0.79**<br>**(-5.74, 3.86)**	CON: -1.66<br>(-8.66, 4.29)<br>DRY: -0.78<br>(-5.70, 3.79)
**start of flowering**	CON: 0.05<br>(0.00, 0.11)<br>DRY: 0.01<br>(-0.04, 0.05)	CON: 0.14<br>(-0.13, 0.42)<br>DRY: 0.04<br>(-0.27, 0.35)	**CON: 0.09**<br>**(-0.02, 0.23)**<br>**DRY: 0.01**<br>**(-0.10, 0.12)**	CON: 0.09<br>(-0.02, 0.23)<br>DRY: 0.01<br>(-0.10, 0.13)

Table S20: *Trifolium pratense* – Selection gradients β for all prior specifications (choice in bold): Posterior mean MCMC estimates for both treatments (CON & DRY), with 95% Bayesian Credible intervals in brackets. Significant selection gradients coloured in blue.

***Trifolium pratense* β**	G10vp2	G10vp2.2	**G10e2**	G10e3
**biomass**	CON: 0.01<br>(-0.02, 0.03)<br>DRY: 0.06<br>(0.00, 0.13)	CON: 0.00<br>(-0.12, 0.12)<br>DRY: 0.12<br>(-0.08, 0.30)	**CON: 0.00**<br>**(-0.05, 0.05)**<br>**DRY: 0.06**<br>**(-0.04, 0.17)**	CON: 0.00<br>(-0.05, 0.05)<br>DRY: 0.01<br>(-0.04, 0.17)
**leaf length**	CON: -0.03<br>(-0.05, -0.01)<br>DRY: -0.05<br>(-0.08, -0.02)	CON: -0.06<br>(-0.15, 0.02)<br>DRY: -0.09<br>(-0.19, 0.02)	**CON: -0.05**<br>**(-0.10, -0.00)**<br>**DRY: -0.06**<br>**(-0.13, 0.00)**	CON: -0.05<br>(-0.10, 0.00)<br>DRY: -0.06<br>(-0.13, 0.00)
**leaf width**	CON: -0.01<br>(-0.04, 0.02)<br>DRY: -0.03<br>(-0.08, 0.02)	CON: 0.01<br>(-0.14, 0.17)<br>DRY: -0.01<br>(-0.18, 0.17)	**CON: 0.00**<br>**(-0.07, 0.08)**<br>**DRY: 0.00**<br>**(-0.10, 0.10)**	CON: 0.00<br>(-0.07, 0.08)<br>DRY: 0.00<br>(-0.10, 0.10)
**SLA**	CON: 0.00<br>(-0.02, 0.02)<br>DRY: -0.01<br>(-0.04, 0.02)	CON: -0.01<br>(-0.10, 0.09)<br>DRY: -0.02<br>(-0.17, 0.13)	**CON: -0.01**<br>**(-0.11, 0.07)**<br>**DRY: -0.04**<br>**(-0.22, 0.08)**	CON: -0.01<br>(-0.12, 0.07)<br>DRY: -0.04<br>(-0.21, 0.08)
**C:N ratio**	CON: 0.01<br>(-0.06, 0.09)<br>DRY: 0.04<br>(-0.08, 0.16)	CON: -0.02<br>(-0.37, 0.35)<br>DRY: 0.04<br>(-0.52, 0.60)	**CON: 0.02**<br>**(-0.14, 0.17)**<br>**DRY: 0.09**<br>**(-0.43, 0.89)**	CON: 0.01<br>(-0.15, 0.18)<br>DRY: 0.15<br>(-0.42, 0.93)
**hair density**	CON: 0.00<br>(-0.09, 0.09)<br>DRY: 0.00<br>(-0.10, 0.10)	CON: 0.00<br>(-0.41, 0.41)<br>DRY: -0.06<br>(-0.32, 0.21)	**CON: -0.01**<br>**(-0.15, 0.13)**<br>**DRY: -0.03**<br>**(-0.15, 0.09)**	CON: -0.01<br>(-0.15, 0.13)<br>DRY: -0.03<br>(-0.15, 0.09)
**plant height**	CON: -0.01<br>(-0.02, 0.01)<br>DRY: -0.01<br>(-0.03, 0.02)	CON: -0.01<br>(-0.09, 0.07)<br>DRY: -0.02<br>(-0.13, 0.09)	**CON: -0.09**<br>**(-0.70, 0.58)**<br>**DRY: -0.03**<br>**(-0.28, 0.12)**	CON: -0.33<br>(-0.71, 0.53)<br>DRY: -0.06<br>(-0.28, 0.11)
**plant width**	CON: 0.01<br>(0.00, 0.02)<br>DRY: 0.02<br>(0.00, 0.04)	CON: 0.01<br>(-0.02, 0.04)<br>DRY: 0.01<br>(-0.08, 0.11)	**CON: 0.01**<br>**(-0.01, 0.03)**<br>**DRY: 0.03**<br>**(-0.05, 0.13)**	CON: 0.01<br>(-0.01, 0.03)<br>DRY: 0.03<br>(-0.05, 0.13)
**start of flowering**	CON: 0.00<br>(-0.02, 0.01)<br>DRY: -0.01<br>(-0.03, 0.00)	CON: -0.01<br>(-0.06, 0.05)<br>DRY: -0.02<br>(-0.05, 0.02)	**CON: 0.00**<br>**(-0.02, 0.01)**<br>**DRY: -0.01**<br>**(-0.03, 0.01)**	CON: 0.00<br>(-0.02, 0.01)<br>DRY: -0.01<br>(-0.02, 0.01)

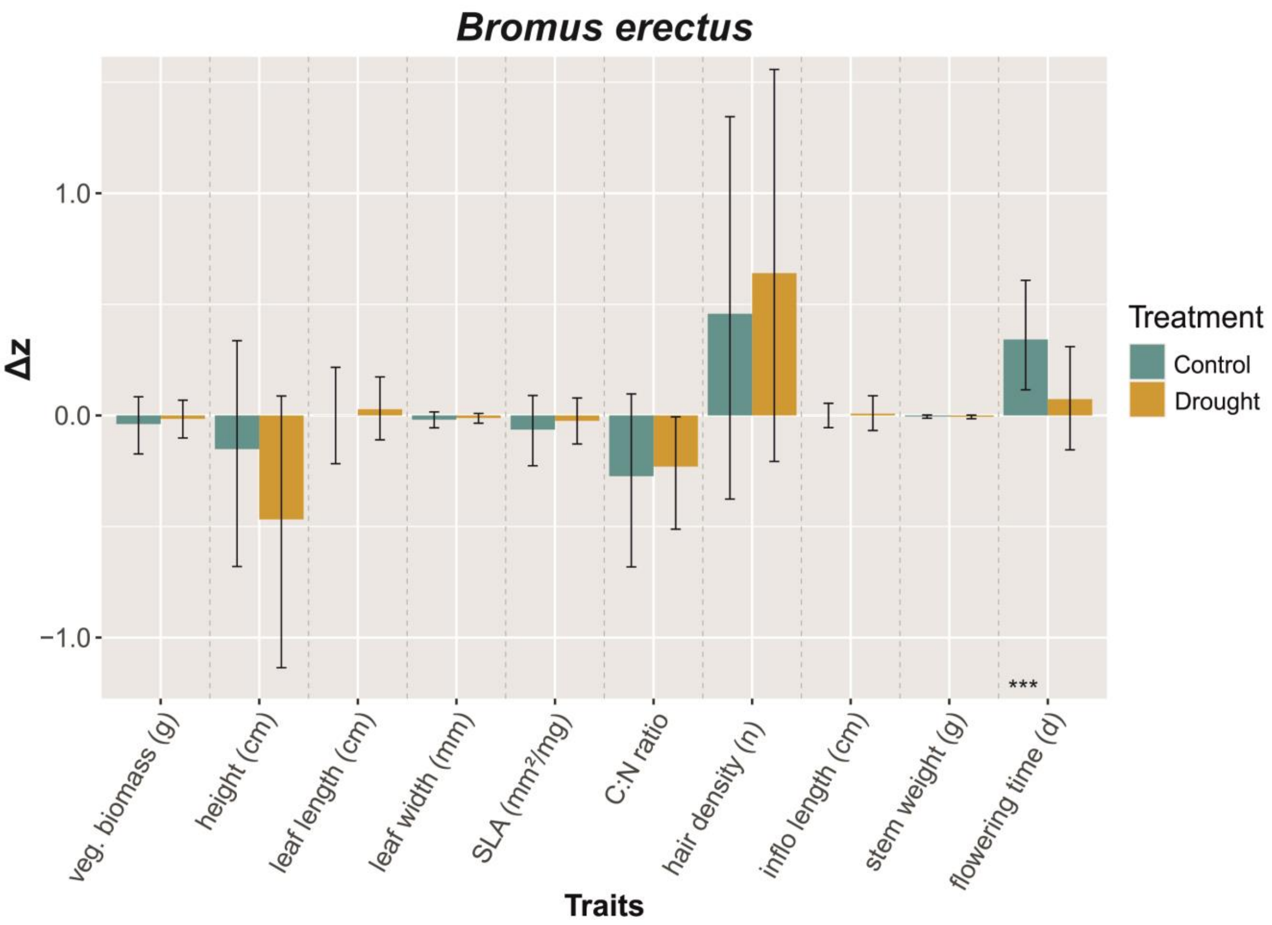
Bromus erectus
Δz
1.0
0.0
−1.0
***
veg. biomass (g)
height (cm)
leaf length (cm)
leaf width (mm)
SLA (mm²/mg)
C:N ratio
hair density (n)
inflo length (cm)
stem weight (g)
flowering time (d)
Traits
Treatment
Control
Drought

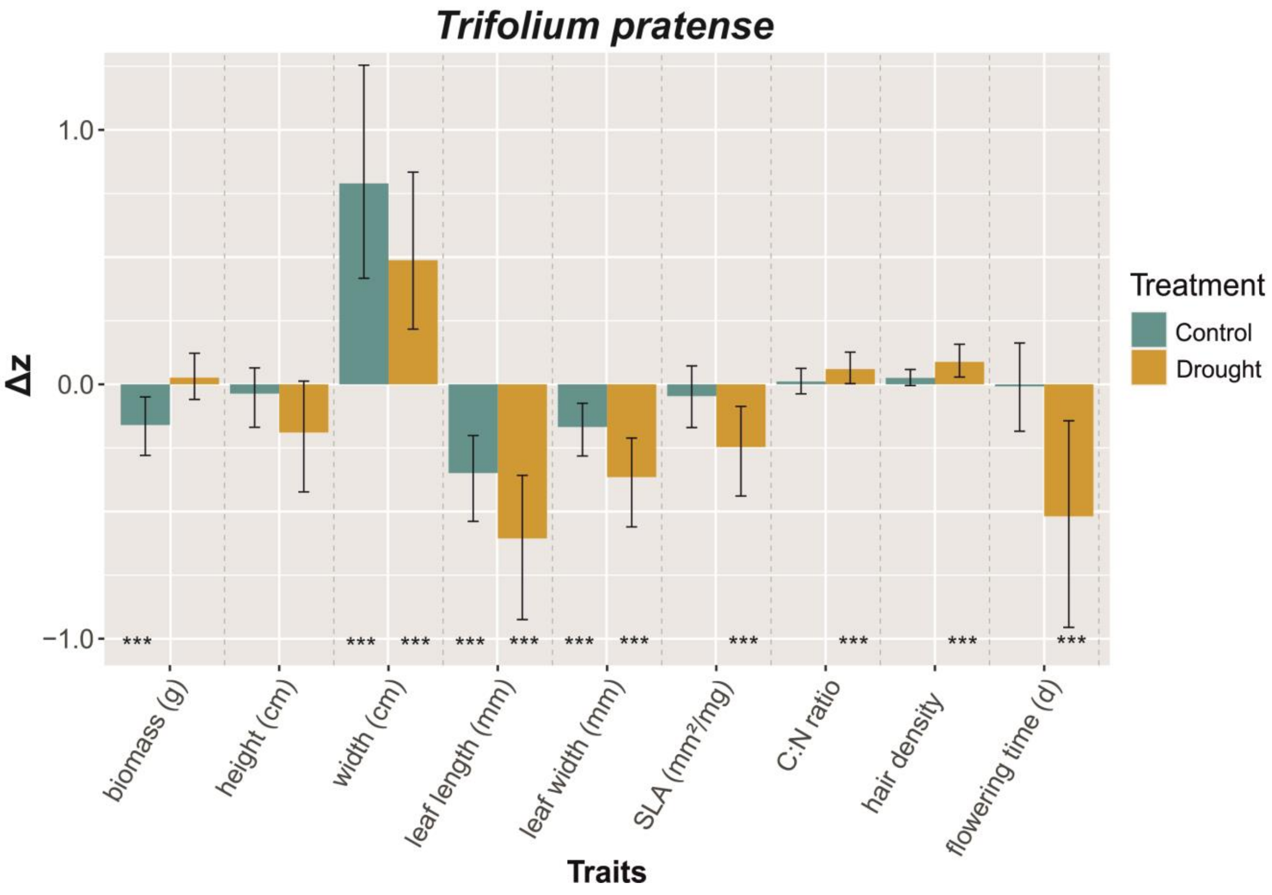
Trifolium pratense
Δz
1.0
0.0
−1.0
*** *** *** *** *** *** *** *** *** *** ***
biomass (g)
height (cm)
width (cm)
leaf length (mm)
leaf width (mm)
SLA (mm²/mg)
C:N ratio
hair density
flowering time (d)
Traits
Treatment
Control
Drought

Figure S1: Response to selection Δz for *Bromus erectus* and *Trifolium pratense*. Quantitative traits are listed on the x-axis. The bar plots for each trait indicate effect sizes for the different treatments, where 'Control' and 'Drought' are coloured in blue and yellow, respectively. 95% Bayesian credible intervals (CI) are shown as measure of uncertainty. #: significant response to selection (CI does not overlap with zero); ***: significant treatment effect (CI does not overlap with other treatment mean); n.s.: no significant treatment effect.
